# Study protocol for a randomized controlled trial of *Pro*portional Assist Ventilation for Mini*mizing* the Duration of Mechanical Ventilation: the PROMIZING study

**DOI:** 10.1186/s13063-023-07163-w

**Published:** 2023-03-27

**Authors:** Karen J. Bosma, Claudio M. Martin, Karen E. A. Burns, Jordi Mancebo Cortes, Juan Carlos Suárez Montero, Yoanna Skrobik, Kevin E. Thorpe, Andre Carlos Kajdacsy-Balla Amaral, Yaseen Arabi, John Basmaji, Gaëtan Beduneau, Francois Beloncle, Guillaume Carteaux, Emmanuel Charbonney, Alexandre Demoule, Martin Dres, Vito Fanelli, Anna Geagea, Ewan Goligher, François Lellouche, Tommaso Maraffi, Alain Mercat, Pablo O. Rodriguez, Jason Shahin, Stephanie Sibley, Savino Spadaro, Katerina Vaporidi, M. Elizabeth Wilcox, Laurent Brochard, On behalf of the Canadian Critical Care Trials Group and, the REVA Network

**Affiliations:** 1 Division of Critical Care, Department of Medicine, Schulich School of Medicine and Dentistry, University of Western Ontario, London, ON Canada; 2 Lawson Health Research Institute, London Health Sciences Centre, London, ON Canada; 3 Interdepartmental Division of Critical Care, University of Toronto, Toronto, ON Canada; 4 Division of Critical Care, Unity Health Toronto - St. Michael’s Hospital, Toronto, ON Canada; 5 Intensive Care Department, Hospital Universitari Sant Pau, Barcelona, Spain; 6 Department of Medicine, McGill University, Québec, Canada; 7 Dalla Lana School of Public Health, Biostatistics Division, University of Toronto, Toronto, ON Canada; 8 Applied Health Research Centre (AHRC), Li Ka Shing Knowledge Institute of St. Michael’s Hospital, Toronto, Canada; 9 Department of Critical Care Medicine, Sunnybrook Health Sciences Centre, 2075 Bayview Ave, Toronto, ON Canada; 10 Intensive Care Department, King Abdulaziz Medical City, Riyadh, Kingdom of Saudi Arabia; 11 Medical Intensive Care Unit, Normandie Univ, UNIROUEN, EA 3830, Rouen University Hospital, 76000 Rouen, France; 12 Medical Intensive Care Department, Angers University Hospital, Angers, France; 13 Service de Médecine Intensive Réanimation, Assistance Publique-Hôpitaux de Paris, CHU Henri Mondor-Albert Chenevier, Creteil, France; 14 Centre Hospitalier de l’Université de Montréal (CHUM) and Hôpital du Sacré-Coeur de Montréal, Montreal, QC Canada; 15 Service de Médecine intensive – Réanimation Département, Hôpital Universitaire Pitié-Salpêtrière and Sorbonne Université Médecine, Paris, France; 16 Department of Surgical Sciences, University of Turin, Turin, Italy; 17 Department of Anaesthesia, Critical Care and Emergency - Città della Salute e della Scienza Hospital – University of Turin, Turin, Italy; 18 Division of Critical Care Medicine, Department of Medicine, North York General Hospital, Toronto, ON Canada; 19 Department of Medicine, Toronto General Hospital, Toronto, ON Canada; 20 Centre de recherche de l’Institut Universitaire de Cardiologie et de Pneumologie de Québec (IUCPQ) – Université Laval, Québec City, QC Canada; 21 Intensive Care Unit, Hôpital Intercommunal de Créteil, Créteil, France; 22 Intensive Care Unit, Instituto Universitario CEMIC (Centro de Educación Médica e Investigaciones Clínicas “Norberto Quirno”), Av. Cnel. Diaz 2423 3rd floor, Buenos Aires, Argentina; 23 Department of Critical Care, Division of Pulmonary Medicine, McGill University, Québec, Canada; 24 Department of Emergency Medicine and Department of Critical Care Medicine, Queen’s University, Kingston, ON Canada; 25 Department of Translational Medicine, Faculty of Medicine and Surgery, University of Ferrara, Ferrara, Italy; 26 University Hospital of Heraklion, Voutes, Heraklion Greece; 27 University Health Network , Toronto, ON Canada; 28 Keenan Research Centre, Department of Critical Care, St Michael’s Hospital, Unity Health Toronto, Toronto, Canada

**Keywords:** Proportional assist ventilation, Work of breathing, Weaning from mechanical ventilation, Patient-ventilatory synchrony, Ventilator-free days

## Abstract

**Background:**

Proportional assist ventilation with load-adjustable gain factors (PAV+) is a mechanical ventilation mode that delivers assistance to breathe in proportion to the patient’s effort. The proportional assistance, called the gain, can be adjusted by the clinician to maintain the patient’s respiratory effort or workload within a normal range. Short-term and physiological benefits of this mode compared to pressure support ventilation (PSV) include better patient-ventilator synchrony and a more physiological response to changes in ventilatory demand.

**Methods:**

The objective of this multi-centre randomized controlled trial (RCT) is to determine if, for patients with acute respiratory failure, ventilation with PAV+ will result in a shorter time to successful extubation than with PSV. This multi-centre open-label clinical trial plans to involve approximately 20 sites in several continents. Once eligibility is determined, patients must tolerate a short-term PSV trial and either (1) not meet general weaning criteria or (2) fail a 2-min Zero Continuous Positive Airway Pressure (CPAP) Trial using the rapid shallow breathing index, or (3) fail a spontaneous breathing trial (SBT), in this sequence. Then, participants in this study will be randomized to either PSV or PAV+ in a 1:1 ratio. PAV+ will be set according to a target of muscular pressure. The weaning process will be identical in the two arms. Time to liberation will be the primary outcome; ventilator-free days and other outcomes will be measured.

**Discussion:**

Meta-analyses comparing PAV+ to PSV suggest PAV+ may benefit patients and decrease healthcare costs but no powered study to date has targeted the difficult to wean patient population most likely to benefit from the intervention, or used consistent timing for the implementation of PAV+. Our enrolment strategy, primary outcome measure, and liberation approaches may be useful for studying mechanical ventilation and weaning and can offer important results for patients.

**Trial registration:**

ClinicalTrials.gov NCT02447692. Prospectively registered on May 19, 2015.

**Supplementary Information:**

The online version contains supplementary material available at 10.1186/s13063-023-07163-w.

## Administrative information

Note: the numbers in curly brackets in this protocol refer to SPIRIT checklist item numbers. The order of the items has been modified to group similar items (see http://www.equator-network.org/reporting-guidelines/spirit-2013-statement-defining-standard-protocol-items-for-clinical-trials/).

**Table:** 

Title {1}	Study protocol for a randomized controlled trial of **Pro**portional Assist Ventilation for Mini**mizing** the Duration of Mechanical Ventilation: The PROMIZING Study
Trial registration {2a and 2b}.	ClinicalTrials.gov Identifier: NCT02447692. https://clinicaltrials.gov/ct2/show/NCT02447692 The protocol is registered on clinicaltrials.gov as NCT02447692, and protocol modifications are shown on clinicaltrials.gov. Additional file 3 shows the main changes which have been made to the protocol and the rationale since the initial registration. All items from the World Health Organization Trial Registration Data Set are included in the trial registry.
Protocol version {3}	PROMIZING Protocol Version 5.0 – December 1, 2019
Funding {4}	This study protocol has been peer reviewed and funded twice by the Canadian Institutes of Health Research (CIHR) through a CIHR Project Grant (2018) and by a CIHR Operating Grant: Industry-partnered Collaborative Research Grant (2014), with Covidien LP, a Medtronic company, as the industry partner. Funding from Covidien LP, a Medtronic company, is provided through the Covidien Investigator Sponsored Research (ISR) Program and subsequently the Medtronic External Research Program (ERP). Covidien LP, a Medtronic company, has also provided in-kind support (loaning of ventilators to sites on an as needed basis to be used only for the purpose of the PROMIZING Study). The funding sources do not have any role in the study design, data collection, analysis, interpretation or publication of manuscript.
Author details {5a}	Dr. Karen J. Bosma* Division of Critical Care, Department of Medicine, Schulich School of Medicine and Dentistry, Western University, and Lawson Health Research Institute, London, Ontario, Canada. KarenJ.Bosma@lhsc.on.ca (Principal author) Dr. Claudio Martin* Division of Critical Care, Department of Medicine, Schulich School of Medicine and Dentistry, Western University, and Lawson Health Research Institute, London, Ontario, Canada. Claudio.Martin@lhsc.on.ca (Principal author) Dr. Karen E. A Burns Interdepartmental Division of Critical Care, University of Toronto Toronto, Canada and Division of Critical Care, Unity Health Toronto - St. Michael’s Hospital, Toronto, Ontario, Canada. Karen.Burns@unityhealth.to Dr. Jordi Mancebo, Intensive Care Department, Hospital Universitari Sant Pau, Barcelona, Spain; jmancebo@santpau.cat Dr. Juan Carlos Suárez Montero, Intensive Care Department, Hospital Universitari Sant Pau, Barcelona, Spain. jcsmjuan2@gmail.com Dr. Yoanna Skrobik, Department of Medicine, McGill University, Québec, Canada. yoanna.skrobik@mcgill.ca Prof. Kevin E. Thorpe, Dalla Lana School of Public Health, Biostatistics Division, University of Toronto, Applied Health Research Centre, St. Michael’s Hospital, Toronto, Ontario Canada. kevin.thorpe@utoronto.ca Dr. Andre Carlos Kajdacsy-Balla Amaral, Interdepartmental Division of Critical Care, University of Toronto and Department of Critical Care Medicine, Sunnybrook Health Sciences Centre, 2075 Bayview Ave, Toronto, Ontario, Canada; andrecarlos.amaral@sunnybrook.ca Dr. Yaseen Arabi, Intensive Care Department, King Abdulaziz Medical City, Kingdom of Saudi Arabia. yaseenarabi@yahoo.com Dr. John Basmaji, Division of Critical Care, Department of Medicine, Schulich School of Medicine and Dentistry, Western University, London, Ontario, Canada. John.basmaji@lhsc.on.ca Dr. Gaëtan Beduneau, Medical Intensive Care Unit, Normandie Univ, UNIROUEN, EA 3830, Rouen University Hospital, 76000, Rouen, France. gaetan.beduneau@chu-rouen.fr) Dr. Francois Beloncle, Medical Intensive Care Department, Angers University Hospital, Angers, France. francois.beloncle@univ-angers.fr Dr. Guillaume Carteaux, Intensive Medicine Resuscitation Department, Henri Mondor University Hospital, Creteil, France. guillaume.carteaux@aphp.fr Dr. Emmanuel Charbonney, Centre Hospitalier de l’Université de Montréal (CHUM) and Hôpital du Sacré-Coeur de Montréal, Montreal, Québec, Canada; emmanuel.charbonney@umontreal.ca Dr. Alexandre Demoule, Service de Pneumologie, Médecine Intensive et Réanimation, Hôpital Universitaire Pitié-Salpêtrière and Sorbonne Université Médecine, Paris, France. alexandre.demoule@aphp.fr Dr. Martin Dres, Service de Médecine intensive – Réanimation Département, Hôpital Universitaire Pitié-Salpêtrière and Sorbonne Université Médecine, Paris, France. martin.dres@aphp.fr Dr. Vito Fanelli, Department of Surgical Sciences, University of Turin, Italy. Department of Anaesthesia, Critical Care and Emergency - Città della Salute e della Scienza Hospital – University of Turin, Italy. vito.fanelli@unito.it Dr. Anna Geagea, MD, FRCPC, Division of Critical Care Medicine, Department of Medicine, North York General Hospital, Toronto, Ontario, Canada. Anna.Geagea@nygh.on.ca Dr. Ewan Goligher, Department of Medicine, Toronto General Hospital, Toronto, ON, Canada; Interdepartmental Division of Critical Care Medicine, University of Toronto, Toronto, Ontario, Canada. Ewan.Goligher@uhn.ca Dr. François Lellouche, Institut Universitaire de Cardiologie et de Pneumologie de Québec (IUCPQ), Québec City, Québec, Canada. Francois.Lellouche@criucpq.ulaval.ca Dr. Tommaso Maraffi, Intensive Care Unit, Hôpital Intercommunal de Créteil, Créteil, France. Tommaso.Maraffi@chicreteil.fr Dr. Alain Mercat, Medical Intensive Care Department, Angers University Hospital, Angers, France. alain.mercat@univ-angers.fr Dr. Pablo Rodriguez, Center for Medical Education and Clinical Research (CEMIC) “Noberto Quirno”, Department of Medicine, Instituto Universitario CEMIC, Buenos Aires, Argentina. pablorodrig16@gmail.com Dr. Jason Shahin, Department of Critical Care, Division of Pulmonary Medicine, McGill University, Québec, Canada. jason.shahin@gmail.com Dr. Stephanie Sibley, Department of Emergency Medicine and Department of Critical Care Medicine, Queen’s University, Kingston, Ontario, Canada. Stephanie.Sibley@kingstonhsc.ca Dr. Savino Spadaro, Department of Translational Medicine, Faculty of Medicine and Surgery, University of Ferrara, Italy. savinospadaro@gmail.com Dr. Katerina Vaporidi, University Hospital of Heraklion, Voutes, Heraklion, Greece. vaporidi@gmail.com Dr. Elizabeth M. Wilcox, University Health Network and University of Toronto, Toronto, Ontario, Canada. Elizabeth.wilcox@utoronto.ca Dr. Laurent Brochard, MD, Keenan Research Centre, Department of Critical Care, St Michael’s Hospital, Toronto, Canada; Interdepartmental Division of Critical Care Medicine, University of Toronto, Toronto, Ontario, Canada. Laurent.brochard@unityhealth.to (Senior Author)
Name and contact information for the trial sponsor {5b}	This is an investigator-sponsored study. The co-principal sponsor-investigators are: Dr. Karen J. Bosma, KarenJ.Bosma@lhsc.on.ca. Dr. Laurent Brochard, Laurent.brochard@unityhealth.to
Role of sponsor {5c}	The study co-principal sponsor- investigators have ultimate authority in study design; collection, management, analysis, and interpretation of data; writing of the report; and the decision to submit the report for publication. The study funders do not have any role in the study design, data collection, analysis, interpretation or publication of manuscript.

## Introduction

### Background and rationale {6a}

Patients with acute respiratory failure (ARF) require invasive mechanical ventilation (MV) to support their work of breathing and provide adequate gas exchange until they recover from their acute illness. Although clinicians aim to wean and liberate patients from MV as soon as patients are capable of breathing unsupported, MV itself may induce respiratory muscle weakness [1–3] and patient-ventilator dyssynchrony [4, 5] and necessitate the administration of sedative drugs, all of which have been associated with a prolonged duration of dependence on MV. Since prolonged invasive ventilation is associated with increased morbidity and mortality [6–8], a major goal is to minimize the duration of weaning and aim for the highest proportion of patients successfully liberated from MV [9]. Avoidance of respiratory muscle atrophy, patient-ventilator dyssynchrony, and heavy sedation may enable clinicians to achieve this goal. Ideally, minimizing respiratory muscle atrophy and patient-ventilator dyssynchrony should theoretically occur if the level of ventilator assistance is adjusted to target normal or reasonable levels of respiratory effort [4, 5, 10].

**Fig. 1:**
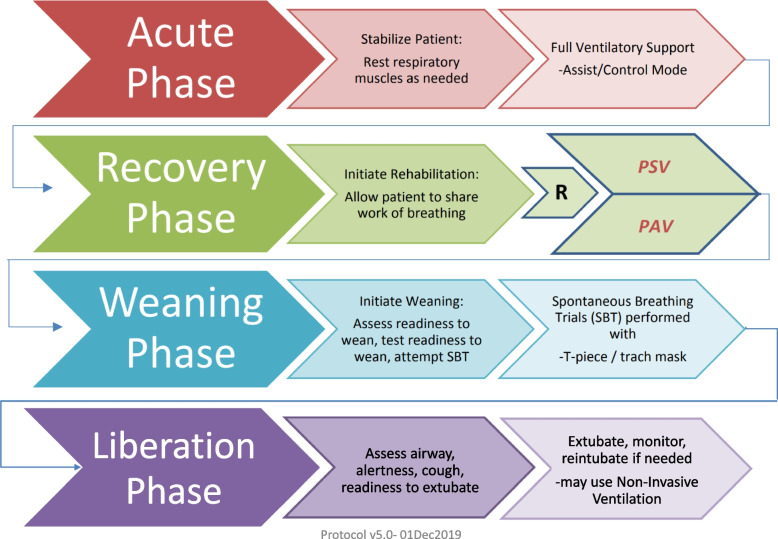
Phases of mechanical ventilation

Proportional assist ventilation with load-adjustable gain factors is a mechanical ventilation mode (PAV+ mode) that delivers assistance to breathe in proportion to the patient’s effort [11, 12]. The proportional assistance, called the gain, can be adjusted by the clinician to maintain the patient’s respiratory effort or workload within a reasonable range. PAV+ mode is the only ventilation mode that allows measurement and targeting of a specific range of respiratory muscle activity by the patient [13]. Currently, pressure support ventilation (PSV) mechanical ventilation mode is the most common mode used after the acute phase of illness [14] and thus may be considered the standard of care for assisted breathing of patients during the recovery phase of acute respiratory failure [15] (Fig. 1). Several small studies have shown short-term advantages of PAV+ over PSV, including improved patient-ventilator synchronization, improved adaptability to changes in patient effort, and improved sleep quality [4, 12, 16–19]. One randomized controlled trial (RCT) compared PAV+ mode to PSV over 48 h and demonstrated it was better tolerated [20], but to date, there has been no large, multi-centre RCT comparing the two modes head to head to evaluate impact on clinically important, patient-centred outcomes.

### Objectives {7}

The objective of this multi-centre RCT is to determine if, for patients with ARF, ventilation with PAV+ mode, instituted early in the recovery phase and set to maintain a workload of breathing within the normal range, will result in a shorter time to successful liberation from invasive MV than with PSV. The secondary objective of the study is to determine if other clinically important outcomes such as ventilator-free days, time from randomization to live hospital discharge, and mortality are better with PAV+ mode as compared to PSV.

### Trial design {8}

The PROMIZING study is a multi-centre, randomized, parallel assignment, open-label clinical trial designed with a superiority framework. Neither the clinical team nor the study investigators will be blinded to the study intervention. The study statistician will be blind to the study arm. Allocation ratio is 1:1 to the two arms of the study.

## Methods: participants, interventions, and outcomes

### Study setting {9}

This multi-centre open-label RCT plans to involve approximately 20 intensive care units (ICUs) in academic and community hospitals located in Canada, France, Italy, Spain, Saudi Arabia, Greece, and Argentina. A list of study sites can be obtained on the PROMIZING study website, www.promizingstudy.com, and on ClinicalTrials.gov (NCT02447692). Ethics approval for the study was obtained from the appropriate research ethics board (REB) for each participating centre.

### Eligibility criteria {10}

#### Run-in phase: identification of sites eligible to participate in the randomization phase

All centres will first undergo a run-in “training” phase with two patients, each assigned to one of the two study arms. The PAV+ run-in patient will receive the study intervention and follow-up until day 7 or until study completion, whichever comes first. The PSV run-in patient will receive the study intervention and follow-up until day 3, or until completing the study, whichever comes first. Upon completion of the run-in patients, the coordinating centre will evaluate the completed Case Report Forms (CRFs) to assess (i) the participating centre’s ability to enroll patients, (ii) compliance with study procedures, and (iii) data completeness and timeliness of data collection. If any problems are identified, further run-in patients will be enrolled, and the centre’s performance re-evaluated. Once the run-in phase is complete, sites will be considered able to enroll and randomize patients in the PROMIZING trial.

#### Eligibility criteria and participant identification

**Fig. 2:**
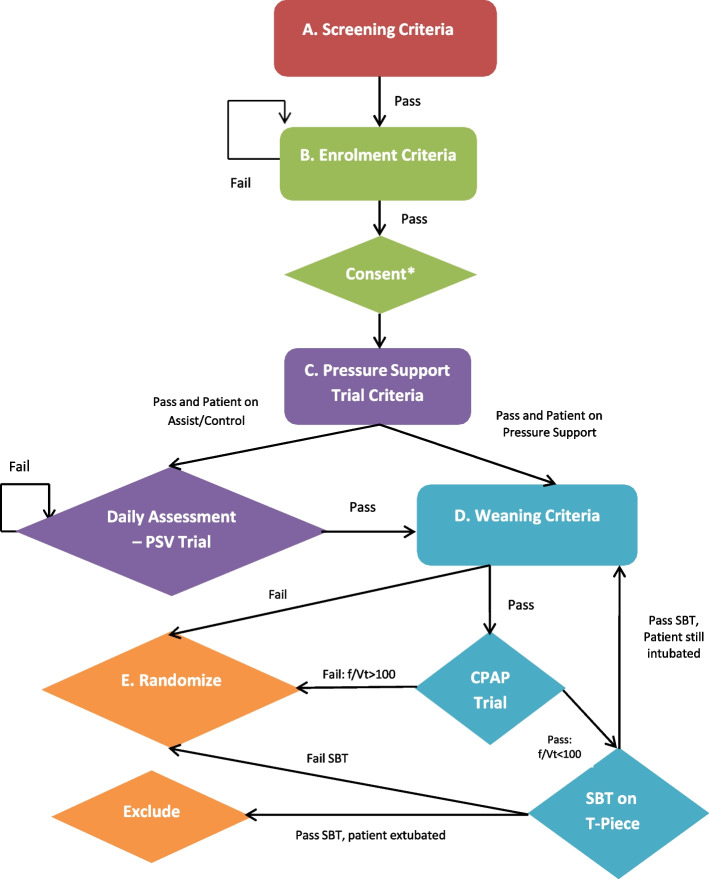
Enrolment algorithm

We will include critically ill adult patients (age ≥18 years) receiving invasive MV for ARF for at least 24 h, and judged ready to commence, and be maintained with, partial ventilatory support (tolerating PSV for at least 30 min) [20] but not yet ready for extubation (not yet ready for a spontaneous breathing trial (SBT) or having failed an SBT). A research coordinator at each study centre will screen patients for eligibility daily. We aim to enroll patients as they enter the recovery phase of their illness (see Fig. 1, with summarized MV modes and MV goals throughout phases of critical illness). We will use a staged recruitment process to identify which eligible patients are enrolled and subsequently randomized in the study if they are not found to be ready for extubation [21]. The five stages, which are performed to ensure that the patient is ready to tolerate PSV but not ready for extubation at the time of randomization, are as follows (see Fig. 2):

(A) Screening Criteria
(B) Enrolment Criteria and Obtaining Consent
(C) Pressure Support Criteria and the Pressure Support Tolerance Trial
(D) Weaning Criteria and the Zero CPAP Trial and the SBT
(E) Randomization Criteria

**Fig. 3:**
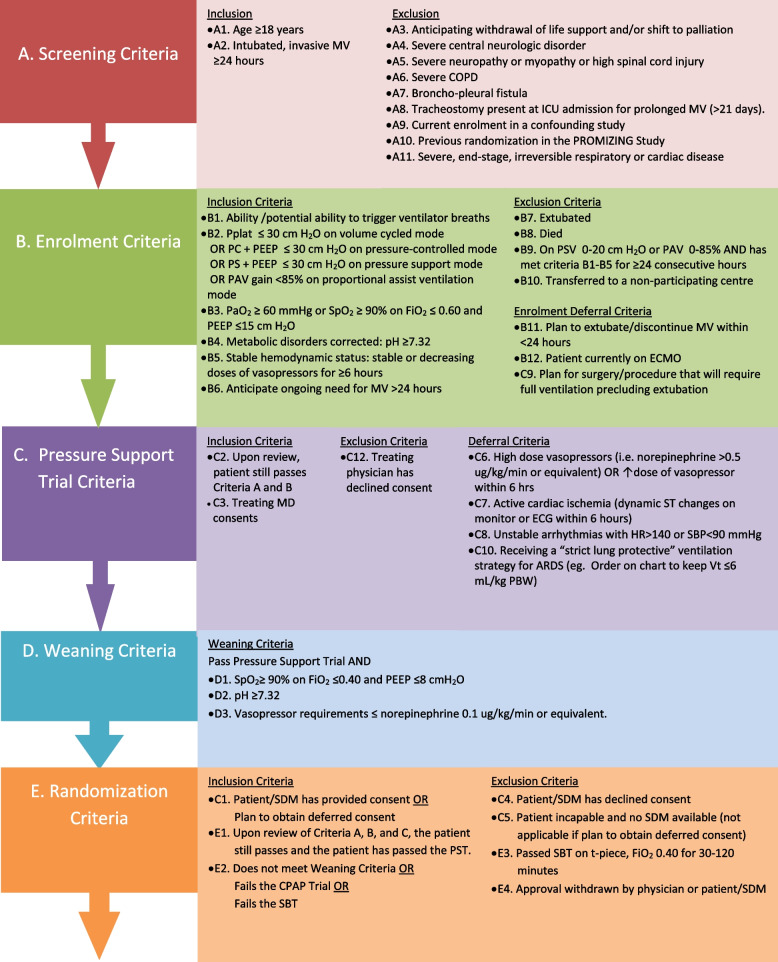
Enrolment criteria

Detailed inclusion and exclusion criteria for each stage are provided in Fig. 3. Participants meeting all of the Screening inclusion criteria and none of the Screening exclusion criteria will be followed daily until they meet the Enrolment Criteria. At that point, consented patients will be enrolled in the study and proceed to the next stage. Enrolled patients meeting Pressure Support Trial inclusion criteria and having no Pressure Support Trial deferral or exclusion criteria will undergo a 30-min Pressure Support Tolerance Trial on pressure support of 5–20 cmH_2_O above positive end-expiratory pressure (PEEP), after which time a blood gas will be drawn. Patient who pass the Pressure Support Tolerance Trial will be assessed for Weaning Criteria. Those meeting all three Weaning Criteria will undergo a 2-min Zero CPAP Trial on 0 cmH_2_O and FiO_2_ 0.40. At the end of 2 min, the frequency to tidal volume ratio will be calculated. Those with a frequency to tidal volume ration <100 will undergo an SBT on t-piece. Patients passing an SBT will be excluded from randomization (enrolled, not randomized). To be randomized, a patient must either fail to meet Weaning Criteria or fail the Zero CPAP Trial or fail the SBT. In summary, patients who satisfy all the inclusion criteria and none of the exclusion criteria, having passed the Pressure Support Trial and either not meeting Weaning Criteria or failing the Zero CPAP Trial or failing the SBT, will be randomized to either PSV or PAV+ in a 1:1 ratio. Further details are provided in Additional file 1.

### Who will take informed consent? {26a}

The participating site principal investigator or their delegate will obtain informed consent with the participant or Substitute Decision Maker (SDM) by means of a dated and signed informed consent form or by witnessed telephone consent. Consent will be obtained prior to randomization whenever possible. However, if the participant is incapable of providing it and the SDM is unavailable within the randomization window, consent will be sought at the earliest moment possible post randomization, and only if the local Research Ethics Board has endorsed deferred consent. The informed consent form must be written in a language that patients and SDMs are familiar with and in accordance with local laws and regulations.

### Additional consent provisions for collection and use of participant data and biological specimens {26b}

Not applicable; no biological specimens will be collected or stored for study purposes, other than bloodwork that is done as part of routine clinical care.

## Interventions

### Explanation for the choice of comparators {6b}

PAV+ is a unique, closed-loop mode of MV that has been shown to optimize patient-ventilator synchrony and patient-ventilator interaction by allowing the clinician to target a normal work of breathing of the patient’s respiratory muscles [13]. The aim of this study is to determine if PAV+ mode is superior to PSV in achieving patient-centred and clinically important outcomes. PSV was chosen as the comparator because it is the most common mode used after the acute phase of illness [14] and thus may be considered the standard of care for assisted breathing of patients during the recovery phase of acute respiratory failure.

### Intervention description {11a}

All patients randomized to PAV+ mode will be treated with ventilators offering this specific mode (Puritan Bennett 840 or 980 series ventilators, Medtronic, Dublin, Ireland). Algorithms for adjusting PAV+ and PSV are provided in the Additional file 2. In brief, the main tool for titrating the gain in PAV+ is the calculation of the muscular pressure generated by the patient that is calculated electronically or using a grid at the bedside. The principle is that the pressure generated by the ventilator above the PEEP is a function of the muscular pressure of the patient and the gain that expresses the percentage of the total pressure. For instance, if the gain is 50%, the pressure of the ventilator and the muscular pressure are equal, sharing the work. If the gain is 70%, the pressure generated by the ventilator is 70% of the total work done by the ventilator and the respiratory muscles. The target for adjusting the gain is to achieve a muscular pressure between 5 and 10 cmH_2_O [13]. It is expected that this setting will make patients comfortable in the majority of cases but special adaptations are proposed for circumstances not covered well by this setting (e.g. major acid-base disturbances, hypoventilation). Patients in the PSV arm may be treated with any ventilator capable of providing PSV. In PSV, settings are according to an algorithm based on current clinical practice (see Additional file 2).

### Criteria for discontinuing or modifying allocated interventions {11b}

**Fig. 4:**
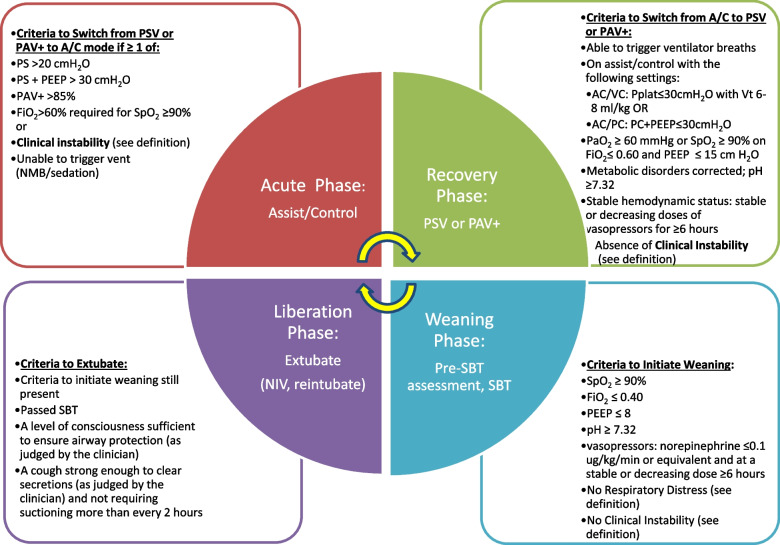
Criteria for transitioning between the acute, recovery, weaning, and liberation phases of mechanical ventilation

In both arms, patients experiencing respiratory distress according to pre-specified criteria will be managed according to recommendations (see PAV+ and PSV algorithms, Additional file 2) but will be returned to assist-control (A/C) mode of ventilation if they require more than 85% of gain in PAV+ or high levels of pressure in PSV (>20 cmH_2_O). In such cases, patients will be assessed at least daily for the transition back to the assigned spontaneous breathing mode and initiating weaning criteria (Fig. 4).

Patients in both arms will be assessed daily for criteria to initiate weaning with the aim of liberating patients from invasive MV at the earliest opportunity. Patients meeting weaning criteria will undergo a 2-min screening test (Pre-SBT readiness assessment) on CPAP of 0 cmH_2_O to determine readiness to proceed to an SBT, which will be conducted on t-piece with FiO_2_ 0.40, or equivalently, connected to ventilator but on zero support for a minimum of 30 min. Patients who pass an SBT will be assessed for extubation based on the level of consciousness and strength of cough as judged by the clinical team.

### Strategies to improve adherence to interventions {11c}

To improve adherence to study interventions, the bedside clinician responsible for making adjustment to the ventilator (e.g. respiratory therapist, physiotherapist, physician) will complete a daily checklist which serves as a reminder and also allows us to document protocol adherence, regarding screening for Criteria to Initiate Weaning, and results of the Pre-SBT readiness assessment and SBTs, as well as use of assist/control mode, PAV+ and PSV throughout the study period, and any deviations from protocol. Additionally, posters are displayed at each bedside as a reminder to the clinicians of the allowed modes of ventilation which may be used according to the treatment arm assignment.

### Relevant concomitant care permitted or prohibited during the trial {11d}

All non-respiratory ICU care will be at the discretion of the treating ICU physician and is non-protocolized; however, we provide guidelines for sedation, nutrition, and early mobilization, and collect data on use of these co-interventions. Guidelines for the administration of sedation and analgesia are provided, with a strong recommendation for using the lowest possible dose of sedating drugs in both arms (or none at all) as required to keep the patient calm and cooperative, avoiding over-sedation whenever possible. When sedation is necessary, we recommend assessing analgesia and intervening with appropriate pharmacological measures prior to administering sedatives. We will record the daily doses of sedatives, analgesics, and neuroleptic medications administered to the patient. Nutrition may be administered enterally or parenterally. Every attempt should be made to ensure patients are receiving adequate calories, protein, and nutrients to meet their energy requirements. We strongly encourage consultation with a registered dietician with experience in critical care whenever possible. Patients should be assessed for ability to participate in passive and active exercises with the aim of mobilizing as early as possible, even while still on the ventilator, as per clinical practice protocols within each ICU. We strongly encourage consultation with a physiotherapist with experience in critical care whenever possible. Further details are available in Additional file 1.

### Provisions for post-trial care {30}

Post-trial care is the responsibility of the treating clinicians. Patients do not receive financial compensation for participating in the study.

### Outcomes {12}

The primary outcome will be the time from randomization to *successful liberation from invasive MV*, which is defined as follows:

1. For patients with an endotracheal tube, “successful liberation” occurs at the time the endotracheal tube is removed (extubation), provided the patient remains alive with no need for reintubation/reinstitution of invasive MV for 7 days post extubation, or until successful ICU discharge, or until live hospital discharge, whichever comes first.
2. For patients with a tracheostomy tube, “successful liberation” occurs at the final time the tracheostomy tube is disconnected from the ventilator, provided the patient remains alive with no need for reinstitution of invasive MV for 7 consecutive days, or until successful ICU discharge, or until live hospital discharge, whichever comes first.

“Successful ICU discharge” is defined as leaving the ICU after liberation from invasive MV, AND remaining alive with no need for reinstitution of invasive MV AND no need for readmission to ICU within 48 h of ICU discharge.

The secondary outcome measures and monitored variables will include:

(i) Ventilator-free days at day 14, 21, and 28 post randomization
(ii) Time from randomization to successful ICU discharge (up to day 90),
(iii) Time from randomization to live hospital discharge (up to day 90),
(iv) Mortality, measured as ICU mortality; hospital mortality; 14-, 21-, 28-, and 90-day mortality
(v) Weaning Progress, measured as time from randomization to: first SBT; first successful SBT; first extubation.
(vi) Weaning Difficulties, measured as the number of patients failing first SBT or first extubation attempt and requiring up to 7 days to extubate (difficult weaning group/group 2); failing first SBT or first extubation attempt and requiring more than 7 days to extubate (prolonged weaning group/group 3),
(vii) Weaning Complications, measured as the number of patients: requiring non-invasive ventilation post extubation; ventilated more than 7 days post randomization, ventilated more than 21 days from time of intubation (prolonged MV group); receiving tracheostomy post randomization, requiring reintubation (up to 7d after planned extubation)
(viii) Safety Endpoint: Frequency and incidence of reported serious adverse events between intervention and control groups
(ix) Co-interventions are also monitored: Tolerance of modes, measured as number of patients ever requiring A/C mode post randomization; number of patient days requiring A/C mode post randomization,
(x) Sedation, measured as cumulative dose of narcotics (converted to morphine equivalents); benzodiazepines (converted to midazolam equivalents); propofol, and dexmedetomidine; antipsychotic medications

“Ventilator-free days” (VFD) are defined as the number of days alive and free of *invasive* ventilation after successful liberation from invasive MV. Non-invasive ventilation may be used after extubation and is not counted as “invasive ventilation.” If the patient dies before achieving successful liberation from invasive MV, that patient will have 0 VFDs. However, if a patient dies AFTER achieving successful liberation, that patient will have the number of VFDs counted as the number of days alive and free of invasive MV occurring between time of successful liberation and time of death. All time intervals and durations will be measured in days (to the nearest 1/10 of a day) and calculated from the day and hour of randomization to the day and hour of the event (e.g. day and hour of successful liberation, successful ICU discharge, live hospital discharge, or death).

The primary and secondary outcomes are clinically relevant and patient-important: prolonged duration of invasive MV is associated with increased morbidity and mortality [6–8], patient discomfort [22, 23], and healthcare costs [24, 25]. Furthermore, requirement for mechanical ventilation of 1 week or more is associated with long-term functional impairment [7, 26], placing a major burden on the healthcare system and significant stress on individual patients and their families [26].

### Participant timeline {13}

**Fig. 5:**
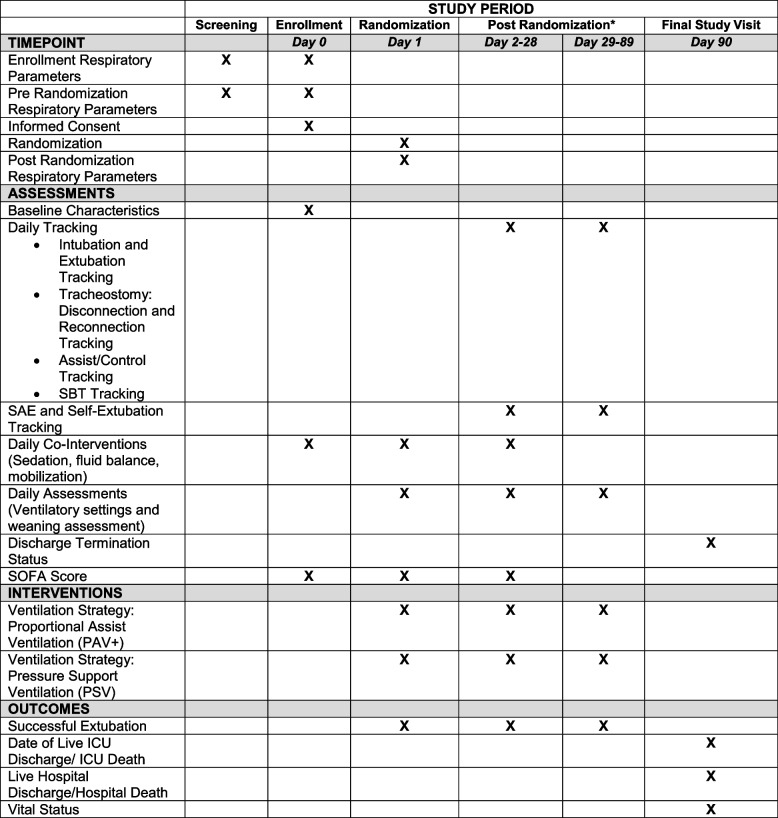
PROMIZING SPIRIT figure. *Data is collected until successful extubation or discharge only

Screening of potential participants begins once ICU patients in participating centres have received invasive MV for at least 24 h. Eligible patients are followed daily until they meet all enrolment criteria, which may occur at any time point, but randomization must occur within 24 h of meeting all enrolment criteria. The study intervention on the assigned mode begins within 60 min of randomization. Participants remain on the assigned ventilator mode algorithm with daily data collection until they achieve successful liberation, death, or 90 days post randomization, whichever comes first (see Fig. 5). Other than determination of vital status at 90 days post randomization, there are no follow-up visits or data collection post ICU discharge.

### Sample size {14}

We used aggregate, blinded data from the first 120 patients to refine our initial sample size calculation (see Additional file 3 for further details). Using a time to event analysis, median time to successful liberation in the entire cohort was 6.8 days. The minimum clinically important difference in time to successful liberation is deemed to be 1.0 day. Using a hazard ratio of 1.30, to demonstrate a reduction in the median duration of ventilation by 1.78 days (assuming 7.70 days versus 5.92), alpha of 0.05 (two-sided), and a power of 80%, requires 529 patients. Anticipating a maximum loss to follow-up (e.g. consent withdrawn, transfer to another hospital) rate of 5%, 558 patients (279 per group) is the minimum number of patients that should be randomized in the study. Using a hazard ratio of 1.25, to demonstrate a reduction in the median duration of ventilation by 1.51 days would require 770 (385 per group) to be randomized in the study (See Additional file 3 for table of calculations). We anticipate being able to enroll a minimum of 558 patients within the planned enrolment period. If enrolment exceeds expectations, we will be powered to show a smaller difference between the 2 groups, which will still be clinically important. The enrolment period will continue until we have complete data on randomized participants and have attained the minimum number of required events in our study cohort.

### Recruitment {15}

Multiple strategies will be employed to achieve adequate participant enrolment to reach at least minimum sample size requirement. We aim to have 20 participating sites in the study. The Run-In phase allows sites and the Coordinating Centre (CC) to determine a site’s capacity to enroll the target one patient/site/month. Participating sites will submit their screening logs to the CC monthly for review and will be contacted by the CC during the study to follow the recruitment and the return of study documents (e.g. copy of screening/enrollment log forms), and to address any issues that the site may have or any questions related to the study. Quarterly meetings will be held with all site investigators and their research teams to provide information sharing from high enrolling centres and address barriers at lower enrolling centres. Additionally, quarterly newsletters will be sent to sites providing updates on enrolment and special recognition to sites receiving certificates of achievement for attaining or surpassing target enrolment each quarter. A study website (promizingstudy.com) will facilitate ease of access to online videos, resources, newsletters and study documents for research teams.

## Assignment of interventions: allocation

### Sequence generation {16a}

Each participant in this study will be randomized to either PSV or PAV+ in a 1:1 ratio in a centralized electronic data capture system called Medidata Rave (Medidata, USA). Participant allocation to treatment will be via variable block randomization with varying block sizes and stratified by site to minimize the likelihood of predicting the next procedure assignment. Randomization will be attained using computer generation sequence methodology, ensuring that the randomization methodology and the generated allocation sequence are concealed from the investigator and participants. The clinical team and the study investigators will know which study intervention is assigned to each patient. The study statistician will be blind to the study arm.

### Concealment mechanism {16b}

Randomization will be attained using computer generation sequence methodology, ensuring that the randomization methodology and the generated allocation sequence are concealed from the investigator and participants.

### Implementation {16c}

Site investigators or their delegates will enter the participant’s entry data into the electronic data capture system (Medidata Rave) to randomize the participant. Using a computer-generated sequence, Medidata Rave will randomize and assign participants to either the PAV+ or the PSV study arm.

## Assignment of interventions: blinding

### Who will be blinded {17a}

The clinical team and the study investigators will know which study intervention is assigned to each patient. The study statistician will be blind to the study arm.

### Procedure for unblinding if needed {17b}

Not applicable; intervention is not blinded.

## Data collection and management

### Plans for assessment and collection of outcomes {18a}

As per accredited ICU standard of care, outcome, baseline, and other trial data are recorded by the clinical ICU team in the patient’s chart or electronic medical record, which will serve as source documents for trial data. Authorized study site personnel designated by the site Investigator (e.g. research coordinator) will collect data from the source documents and enter the data into the electronic case report forms (eCRF). A daily checklist to promote and monitor protocol adherence will be completed by the bedside clinician managing the ventilator and will serve as a source document. All data collection forms can be found on the study website (promizingstudy.com).

### Plans to promote participant retention and complete follow-up {18b}

Since study participants are ICU patients receiving invasive MV, we anticipate retention and complete follow-up in 95% of randomized patients. However, we anticipate a maximum of 5% of patients may be lost to follow-up if they are transferred to another hospital of if they or their SDMs withdraw consent for ongoing study participation. In the event that a participant revokes authorization to collect or use their data, the investigator, by regulation, retains the ability to use all information collected prior to the revocation of participant authorization, unless otherwise indicated by local REB regulations. For participants who have withdrawn consent, attempts should be made to obtain permission to collect at least vital status (i.e. that the participant is alive, which is publicly available data) at the end of their scheduled study period.

### Data management {19}

Medidata RAVE® will be used for this study for web-based randomization and data collection and has detailed quality checks programmed to ensure data integrity. All study data will be entered in electronic case report forms (eCRF) at the study site. Appropriate security measures will be taken to authorize study site personnel using unique usernames and passwords prior to any data being entered into the system. The study data will be housed on a secure in-house server at St. Michael’s Hospital in Toronto, Canada, throughout the duration of the study, and up to 15 years after the study is complete. All eCRF corrections are to be made by an Investigator or other authorized study site personnel. The site Investigator must confirm by his/her electronic signature in a specific section of the eCRF to confirm that he/she has reviewed the data and that the data is complete and accurate. Data validation procedures are described in detail in the Data Validation and Management Plans.

### Confidentiality {27}

Original records for each participant in the study will be maintained in separate files in a secure, limited access location at the study site for the duration of the study and after study completion for 15 years. Copies of the de-identified documents may be made and supplied to the sponsor representative for the purposes of ongoing data monitoring and analysis of results. Participants’ identities will be kept confidential by assigning each participant a participant ID upon enrollment into the study. The investigator must assure that participant confidentiality will be maintained and that participant identities shall be protected from unauthorized parties. On eCRFs or other documents submitted to the CC, participants should not be identified by their names, but by their participant identification code, which is assigned in the eCRF. The investigator should keep a participant code log relating codes to the names of participants. The investigator should maintain study documents that are not for submission to the CC (e.g. participants’ written consent forms), in strict confidence.

### Plans for collection, laboratory evaluation and storage of biological specimens for genetic or molecular analysis in this trial/future use {33}

Not applicable; no biological specimens will be collected or retained for this trial /future use.

## Statistical methods

### Statistical methods for primary and secondary outcomes {20a}

Analysis will follow the intention-to-treat principle. Baseline data will be analysed descriptively (e.g. mean and standard deviation, median and interquartile range, counts and percentages as appropriate). Time to liberation will be summarized by cumulative incidence curves with death treated as a competing risk and compared between groups with the Fine-Gray test. The treatment effect will be expressed as a hazard ratio with 95% confidence interval from a multistate generalization of the Cox model. Additionally, a cause-specific Cox model where death is treated as a censoring event will be fit for comparison. Patients with missing outcome data will be censored at last contact. The secondary outcomes of ventilator-free days will be compared between the groups by means of a Wilcoxon test. The treatment effect will be expressed as the difference in median ventilator-free days along with a 95% confidence interval obtained by bootstrap methods. The time-to-event outcomes ICU discharge and hospital discharge present the same special challenge as the primary outcome because death is a competing risk. To mitigate these problems, cumulative incidence curves will be constructed that provide estimates of the outcome of interest, accounting for death. Cause-specific treatment effects will be given as hazard ratios with 95% confidence intervals from Cox models. Time-to-death will be analysed using standard methods for survival data (Kaplan-Meier curve, log-rank test), and the treatment effect will be expressed as a hazard ratio with 95% confidence interval. Four of the secondary outcomes describe weaning difficulties in various ways. These also suffer from the competing risk of death and other improper subgroup issues. These outcomes will be primarily descriptive. The outcome, “requiring A/C” will be compared by a chi-square test (or Fisher’s exact test) and treatment difference will be expressed as an odds ratio with 95% confidence interval. For days requiring A/C, patients not receiving A/C will be assigned a value of zero. Although a *t*-test is the appropriate parametric approach, the non-parametric Wilcoxon rank-sum will be favoured if there are many “null” (zeros) entries. Total medication dose, pain, sedation/agitation, and delirium scores will be compared by a *t*-test between study arms. Ordinal regression analyses will be used to assess factors associated with weaning group classification (short/difficult/prolonged weaning groups). Exploratory analyses using multiple regressions will be used to identify factors associated with duration of MV.

The secondary outcome of ventilator-free days has several difficulties. Analysis of “survivors only” results in an improper subgroup and potentially loses the benefits of randomization. We therefore plan to conduct secondary and supplemental analyses to better understand any observed treatment effect. One analysis will assign −1 to deaths (instead of zero) to make that distinction. We will also examine time on a ventilator in a few ways, some of which subsume secondary outcomes. A Poisson regression model will be considered, as it takes into account ventilated days as the outcome, with the logarithm of the number of days observed (up to 21) as an offset. Finally, a marginal structural model will be employed; first, the probability of surviving will be modeled; then, the weighted analysis of the survivors will be carried out using the inverse of the survival probability as the weight.

### Interim analyses {21b}

There are no plans to conduct an interim analysis of the outcomes, as per the statistical analysis plan.

### Methods for additional analyses (e.g. subgroup analyses) {20b}

We have planned subgroup analyses based on (a) duration of MV prior to randomization (a duration greater than 5 days is associated with prolonged weaning); (b) failed SBT prior to randomization, depicting a subgroup classified as difficult weaning; (c) failed extubation prior to randomization, depicting a subgroup classified as difficult weaning; (d) mild vs. moderate vs. severe frailty; and (e) positive for COVID-19. A secondary analysis of outcomes will be done with adjustment for covariates (baseline clinical variables specified a priori with potential effect on duration of MV) and clinically important prognostic variables at baseline (e.g., Acute Physiology and Chronic Health Evaluation (APACHE) score). A secondary Bayesian analysis of primary outcome is also planned. We have planned two sensitivity analyses of primary and secondary outcomes, based on (a) defining “successful liberation” as “48 h alive without reinstitution of invasive MV”; and (b) assigning a value of 0 VFDs to any participant who dies at any time during the VFD period.

### Methods in analysis to handle protocol non-adherence and any statistical methods to handle missing data {20c}

Statistical analysis will be based on intention-to-treat, per protocol analysis. Patients with missing outcome data will be censored at last contact. Descriptive statistics on use of non-protocolized modes as well as intolerance of PAV+ and PSV (duration in days and percentage of time in study spent on assist/control mode) will be provided.

### Plans to give access to the full protocol, participant-level data and statistical code {31c}

There are no plans at present in place for granting public access to the full protocol, participant-level dataset and statistical code. Additional secondary analyses, using the database, can be proposed by investigators and will be discussed with the executive steering committee. For 12 months after publication of the final study results, investigators will be given priority to use the data for secondary analyses, after which time requests for access to de-identified data will be considered.

## Oversight and monitoring

### Composition of the coordinating centre and trial steering committee {5d}

Lawson Health Research Institute (Lawson) in London, Canada, will oversee all contracts, trial insurance and financial disbursements. The Applied Health Research Centre (AHRC) in Toronto, Canada, will serve as the Coordinating Centre (CC) for the study. The AHRC research project manager will meet weekly with the co-PIs (KJB, LB) throughout the study. The Executive Steering Committee (EC) will consist of the lead investigators (KJB, LB, KEAB, CM, JM, YS) with support from the study statistician (KT) and the AHRC coordination centre. The EC will oversee all aspects of the study including implementation of all policies and the daily operations. The EC will meet weekly during the planning phase of the trial and alternate months thereafter. Regular meetings with Site Investigators by region (e.g. Europe, Canada, Argentina, Saudi Arabia) will occur before enrollment begins, and monthly or bi-monthly to discuss enrollment rates and non-adherence and at the completion of the study.

### Composition of the data monitoring committee, its role and reporting structure {21a}

The Data Safety and Monitoring Board (DSMB) will act in an advisory capacity to safeguard the interests of study participants and assess the safety (not efficacy) of the interventions during implementation of the multi-centre trial and will monitor the overall conduct of the trial. The DSMB has 3 members with representation from North America and Europe. All members declare no competing interests. The DSMB will review safety reports biannually. The DSMB will have the ability to request additional safety analyses and make recommendations about the safe conduct of the trial. The DSMB Chair will review all serious adverse events classified as probably or definitely related to enrolment in the trial within 7 days and communicate directly with the co-principal investigators, who in turn will communicate back to the Executive Steering Committee. The DSMB is independent of the EC and co-PIs with regard to the recommendations made, but is supportive of the aims and methods of the trial. The DSMB, EC and co-PIs will work collaboratively to ensure rigorous, safe and timely conduct of the trial. Further details can be found in the DSMB Charter (v. March 8, 2016), available from the AHRC or co-PIs upon request.

### Adverse event reporting and harms {22}

Although we do not anticipate any additional risks to be incurred by participating in the study, the following monitoring approach will be employed to ensure safety of patients. All serious or unexpected adverse events with potential causality to the intervention or that are believed to be potentially directly related to enrolment in the study (i.e. it is unlikely to have occurred if the patient were not enrolled in the study) will be reported to the CC and Monitor through the electronic case report form and will be reviewed by the DSMB at biannual meetings. Serious adverse events (SAEs) are to be documented in source and in the serious adverse events eCRF. The investigator or his/her designate will promptly report SAEs with probable or definite relation to study intervention within 24 h of becoming aware of the event, and update as additional information becomes known. Each site will also need to report SAEs with probable or definite relation to a study intervention to the local REB according to local institutional requirements.

### Frequency and plans for auditing trial conduct {23}

A combination of centralized and on-site or virtual monitoring activities will be used to ensure the quality of the data captured, the study operations and the safety of patients. On-site or virtual monitoring visits will be planned by the CC for each site when they have accrued 7–10 patients. A risk-based approach will govern the monitoring activities thereafter. Source data verification on critical data elements will be performed on a selection of the participants by comparing the data in the patient’s files (source documents) with data in the CRF, and will be conducted as per the Sponsor-approved Monitoring and Quality Plan. Electronic CRF will not constitute source documentation and data entered in the CRF must be traceable to an original source record (electronic or paper) either as part of the electronic database or in the patient’s file. For selected patients, the presence of a signed written informed consent as well as compliance with inclusion and exclusion criteria will be checked. The site investigator will permit study-related monitoring, audits, and inspections by the REB, the Sponsor, and the CC of all study-related documents (e.g. source documents, regulatory documents, data collection instruments, study data). The site investigator will ensure the capability for inspections of applicable study-related facilities (e.g. intensive care units).

### Plans for communicating important protocol amendments to relevant parties (e.g. trial participants, ethical committees) {25}

The CC is responsible for the distribution of any protocol amendments to site Investigators. Site Investigators are responsible for the distribution of an amendment to all staff involved in the study and for obtaining approval for the amendment from the local REB as required by local institutional guidelines.

### Dissemination plans {31a}

Since the PROMIZING study is a multi-centre study, it is the intention of the co-Principal Investigators that the first publication or presentation of the results and data of this study will be made to our collaborators in the two scientific networks supporting this study: the Canadian Critical Care Trials Group (CCCTG) and the Réseau Européen de Recherche en Ventilation Artificielle (REVA), and in conjunction with presentation of the study results and data with the investigators and the institutions from all appropriate sites contributing data, analyses and comments. Site principal investigators will be invited to contribute to writing and reviewing the primary manuscripts and abstracts. The primary manuscript and abstracts will undergo review by the steering committee and internal peer review by the CCCTG.

For the main results, we plan to submit an abstract for presentation at an international critical care meeting and possibly to national meetings, and we plan to submit a manuscript for publication in an international medical journal.

## Discussion

Meta-analyses comparing PAV+ to PSV suggest PAV+ may benefit patients and decrease healthcare costs [27–32]. No study to date has targeted the difficult to wean patient population most likely to benefit from the intervention, or used consistent timing for the implementation of PAV+. In this study protocol, we describe a multi-centre, sufficiently powered, randomized clinical study needed to show an impact on clinically important, patient-centred outcomes. We carefully designed an enrollment algorithm to ensure that all patients randomized could not be separated from mechanical ventilation at the time of enrolment. For trials of weaning from mechanical ventilation, the choice of an outcome measure and approaches to separation from the ventilator are important considerations but with no consensus. In the following section, we discuss the rationale for our primary outcome, the definition of the primary outcome, standardization of the weaning method and other unique aspects of this trial protocol.

Regarding the primary outcome, there is no single perfect outcome for this patient population as patients are randomized at various timepoints after ICU admission and this explains the successive approaches we have been taking before and after our analysis of the aggregate, blinded data from the first 120 patients (see Additional file 3). Mortality is a frequent outcome measure for trials in critical care, but for this trial it is not appropriate as the primary outcome as we are not anticipating a mortality benefit but rather a shortening in duration of MV [27–30]. However, death is a competing risk for alternative outcomes such as duration of mechanical ventilation. Ventilator-free days is a composite outcome measure that has been proposed as one approach to account for this [33]. The VFD outcome has several shortcomings [34, 35], one of which is that death is indistinguishable from someone alive but still ventilated; although being dead versus being alive on a ventilator are highly different from a patient and family perspective, the VFD outcome treats them as equivalent [33]. Death can be more heavily weighted in a VFD analysis by assigning a score of −1, but this is arbitrary [35]. With either approach to death, the distribution of VFD can be highly skewed and bimodal, further complicating the analysis and moreover the clinical interpretation. Analysis of survivors only results in an improper subgroup and potentially loses the benefits of balanced confounders due to randomization. We therefore chose time to liberation from mechanical ventilation as the primary outcome for our trial, with death treated and evaluated as a competing risk [36].

Liberation from mechanical ventilation is not a discrete event since extubation failure is common [37]. Extubation failure is defined as the need for reintubation, but the interval before successful liberation is declared varies from 48 h to 1 week [38]. Liberation from mechanical ventilation of patients with tracheostomy can be similarly defined as continuous disconnection from the ventilator. For our trial, we chose the more conservative measure with the requirement to be free of mechanical ventilation support and remaining alive for 7 days.

The decision to extubate a patient or disconnect from the mechanical ventilator in the case of a patient with a tracheostomy, may influence our primary outcome. Since this is not a blinded trial, this is an important potential source of bias. We chose to address this by including recommendations for routine evaluation of weaning readiness and spontaneous breathing trials to guide this decision. We chose to standardize the spontaneous breathing trial by using a t-piece rather than pressure support ventilation. While some publications support a modest benefit for using PSV as the mode of SBT [39, 40], we reasoned that using PSV in the PAV+ group may lead to protocol violations and furthermore may disadvantage the PAV+ group as those patients will experience a change in the mode of support. Using t-piece is a familiar approach that can be consistently used in both groups and provides a reliable measure of readiness for ventilator liberation since it most accurately reflects work of breathing post extubation [41].

Strengths of the study include the multinational participation that will increase the external validity of our results, and the specific enrollment process in the study (five steps) which ensures that all patients still need ventilation. This pre-randomization algorithm meticulously selects patients who tolerate partial ventilatory support but do not strictly reach criteria for ventilator separation. This part is relatively demanding for centres, which represent a relative limitation for enrollment. For this reason, it is expected that some patients may be enrolled but not randomized. Both study arms are guided by well-defined protocols to guide ventilator adjustments, with a physiologic basis for adjusting PAV+ gain setting. Lack of familiarity with PAV+ may be a study limitation. However, most of the participating centres have prior experience with both modes of ventilation, training is provided on the ventilator modes, and a run-in phase is mandatory prior to enrolling patients. The mandatory run-in phase also safeguards the fidelity of the trial, by ensuring centres can deliver the treatments and record the data according to the proposed methods. We use deferred consent to increase enrollment in centres where that is approved by the local research ethics board.

Several challenging aspects were encountered in designing the trial, reflected by the amendments to the protocol. Those challenges included the following: deliberations on the primary outcome for the study (VFDs being considered as poorly patient-centred and having a very skewed distribution); the population(s) selected (avoiding patients with very poor outcome or high likelihood of chronic ventilation and deferring enrolment for patients awaiting surgery or on ECMO); and the consent model utilized (using deferred consent because of the short time window to enroll patients). The resultant version 5.0 of the PROMIZING Study Protocol reflects a study designed to accurately capture the populations of interest at a common timepoint in their critical illness trajectory, enroll and randomize them to well-defined ventilation algorithms, minimize bias, guide co-interventions, and analyse results in a comprehensive, patient-centred manner appropriate for this patient population.

In conclusion, we describe a protocol for a multi-centre RCT in patients who have been receiving invasive MV and who are not yet ready for liberation to compare PAV+ mode to PSV on the time to successful liberation from mechanical ventilation. Our rationale for the primary outcome measure and liberation strategies may be useful for other studies of mechanical ventilation and weaning.

## Trial status

This publication is based on version 5.0 of the PROMIZING study protocol (December 1, 2019). Recruitment began September, 2016, with 2 sites. New participating sites continue to be added. Enrolment was suspended in April, 2020, due to the COVID-19 pandemic and resumed on a site-by-site basis as local restrictions allowed. We anticipate completing recruitment in 2023.

## Supplementary Information

**Additional file 1.** Detailed study methods.

**Additional file 2.** PAV_PSV ventilation algorithms.

**Additional file 3.** Rationale for Changes to Protocol.

## Data Availability

There are no plans at present in place for granting public access to the full protocol, participant-level dataset and statistical code. Additional secondary analyses, using the database, can be proposed by investigators and will be discussed with the executive steering committee. For 12 months after publication of the final study results, investigators will be given priority to use the data for secondary analyses, after which time requests for access to de-identified data will be considered. Requests for data presented in the final paper can be made to the PROMIZING Executive Committee, by way of email to the corresponding authors. Data provided will consist of de-identified participant with data dictionary, restricted to the data presented in the paper. Any data provided will be subject to a data sharing agreement.

## Abbreviations

A/C: Assist/control
AHRC: Applied Health Research Centre
APACHE: Acute Physiology and Chronic Health Evaluation
ARF: Acute respiratory failure
CC: Coordinating centre
CCCTG: Canadian Critical Care Trials Group
CIHR: Canadian Institutes of Health Research
CHUM: Centre Hospitalier de l’Université de Montréal
CPAP: Continuous positive airway pressure
CRFs: Case report forms
DSMB: Data Safety and Monitoring Board
EC: Executive Steering Committee
ECMO: Extracorporeal membrane oxygenation
eCRF: Electronic case report form
ERP: External research programme
ISR: Investigator-sponsored research
ICU: Intensive care unit
IUCPQ: Institut Universitaire de Cardiologie et de Pneumologie de Québec
Lawson: Lawson Health Research Institute
MV: Mechanical ventilation
PAV+: Proportional assist ventilation with load-adjustable gain factors
PEEP: Positive end-expiratory pressure
PI: Principal Investigator
PROMIZING: Proportional Assist Ventilation for Minimizing the Duration of Mechanical Ventilation
PSV: Pressure support ventilation
RCT: Randomized controlled trial
REB: Research Ethics Board
REVA: Réseau Européen de Recherche en Ventilation Artificielle
SAEs: Serious adverse events
SBT: Spontaneous breathing trial
SDM: Substitute decision maker
VFD: Ventilator-free days
