# Supplementary material for: Study protocol for a randomized controlled trial of Proportional Assist Ventilation for Minimizing the Duration of Mechanical Ventilation: the PROMIZING study
Source: Trials. 2023 Mar 27;24:232. doi: 10.1186/s13063-023-07163-w (PMC10041480; doi:10.1186/s13063-023-07163-w)
Supplement: Supplementary file 1 — Additional file 1. Detailed study methods. [file 13063_2023_7163_MOESM1_ESM.pdf]

Detailed Study Methods

Screening Criteria

Patients are screened for eligibility while in the acute phase of respiratory failure according to the following criteria:

Inclusion

1. Age ≥18 years
2. Intubated and receiving any mode of invasive MV ≥24 hours

Exclusion

1. Anticipating withdrawal of life support and/or shift to palliation as the goal of care
2. Severe central neurologic disorder (e.g., Hemorrhage, stroke, tumour) causing elevated intracranial pressure, or impaired control of breathing, or requiring specific ventilator adjustments (i.e. To attain specific CO_2 target) or requiring neurosurgical intervention
3. Known or suspected severe or progressive neuromuscular disorder likely to result in prolonged or chronic ventilator dependence (e.g., Guillain-Barré syndrome, Myasthenia Gravis, ALS, MS, high spinal cord injury, kyphoscoliosis or other restrictive disorder) (Obesity hypoventilation syndrome that may be managed with nocturnal non-invasive ventilation is NOT an exclusion)
4. Severe COPD: Baseline daytime hypercapnea ($pCO_2 > 50$ mmHg) OR GOLD 4 airflow limitation ($FEV_1 < 30\%$ predicted) OR MRC class 4 symptoms ("I am too breathless to leave the house" OR "I am breathless when dressing")
5. Broncho-pleural fistula

6. Tracheostomy present at ICU admission for the purpose of chronic or prolonged mechanical ventilation (>21 days). (A patient who was endotracheally intubated for acute respiratory failure and received a tracheostomy during their ICU admission, prior to enrolment, is not excluded).
7. Current enrolment in a confounding study, as assessed by the steering committee
8. Previous randomization in the PROMIZING Study

9. Severe, end-stage, irreversible respiratory or cardiac disease (e.g. interstitial lung disease, pulmonary fibrosis, cardiomyopathy, valvulopathy) likely to result in prolonged or chronic ventilator dependence /unlikely to wean from mechanical ventilation (Patients who are candidates for intervention to treat the underlying respiratory/cardiac disease (e.g. lung transplant, heart transplant, cardiac surgery) may be re-evaluated once intervention is complete)

Enrolment Criteria

These criteria will be evaluated as patients approach the recovery phase of their illness and consent will be requested if these criteria are met, the treating physician provides consent, and there is no plan to extubate or discontinue mechanical ventilation within 24 hours. Enrolment will be deferred if the patient is currently receiving extracorporeal membrane oxygenation (ECMO) or awaiting surgery or a procedure that would preclude extubation.

Inclusion

1. Ability or potential ability to trigger ventilator breaths (i.e. not receiving neuromuscular blockade).
2. On Assist/Control volume-cycled ventilation: Technically satisfactory plateau pressure ≤ 30 cm H_2O; OR on Assist/Control pressure-controlled ventilation or similar mode: Pressure control plus

PEEP ≤ 30 cm H_2O; OR on Pressure Support ventilation: Pressure support plus PEEP ≤ 30 cm H_2O; OR on Proportional Assist ventilation: PAV gain <85%

3. PaO_2 ≥ 60 mmHg or SpO_2 ≥ 90% on FiO_2 ≤ 0.60 and PEEP ≤ 15 cm H_2O
4. Metabolic disorders corrected: pH ≥7.32
5. Stable hemodynamic status: stable or decreasing doses of vasopressors for ≥6 hours
6. Anticipate ongoing need for ventilation >24 hours

Exclusion

1. Extubated
2. Died
3. Has met Enrolment Inclusion criteria 1-5 AND tolerated pressure support of 0-20 cm H_2O or proportional assist ventilation of 0-85% for ≥24 consecutive hours (including time on CPAP, t-piece, or tracheostomy mask).
4. Patient transferred to a non-participating centre

Pressure Support Criteria and the Pressure Support Tolerance Trial

A patient who has met the screening and enrolment criteria will undergo a pressure support tolerance trial (PSTT) unless any of the following deferral criteria are present:

1. High dose vasopressor requirements (i.e. epinephrine or norepinephrine >0.5 ug/kg/min or equivalent) OR patient requiring an increase in dose of vasopressor within 6 hrs
2. Active cardiac ischemia (dynamic ST changes on monitor or ECG within 6 hours)
3. Unstable arrhythmias with HR>140 or SBP<90 mmHg
4. Receiving a “strict lung protective” ventilation strategy for ARDS

If deferral criteria are present, patients will be reassessed daily. In the absence of deferral criteria, patients will be placed on PSV 10-20 cmH_2O (above PEEP) for at least 30 minutes under direct clinical observation, at the same PEEP and FiO_2 settings they were on prior to the PST. The maximum settings allowed for the PST are a pressure support of 20 cmH_2O or PS+PEEP=30 cmH_2O. The PST can be stopped at any time for Respiratory Distress or Clinical Instability (see definitions below). At the end of 30-120 minutes, an arterial blood gas will be drawn. Patients fail the PST if they develop prolonged apnea, Respiratory Distress, Clinical Instability, or require PS >20 cmH_2O, PS + PEEP > 30 cmH_2O, or require FiO_2>60% to maintain SpO_2>90%, or respiratory acidosis with pH <7.32 despite maximum PS of 20 cmH_2O. Patients who fail the PSTT will be returned to the previous ventilator settings and will be re-evaluated daily provided they still meet all study criteria.

Weaning Criteria and the Zero CPAP Trial and the Spontaneous Breathing Trial (SBT)

Patients who pass the PST will undergo a weaning assesment if they meet all of the following Weaning Criteria:

1. SpO_2≥ 90% on FiO_2 ≤0.40 and PEEP ≤8 cmH_2O
2. pH ≥7.32
3. Vasopressor requirements no higher than norepinephrine 0.1 ug/kg/min or equivalent.

Patients meeting all Weaning Criteria will then undergo a Zero CPAP Trial: the ventilator mode is changed to continuous positive airway pressure (CPAP) at 0 cmH_20 and FiO_2 0.40 for 2 minutes, at which time the frequency to tidal volume ratio (f/Vt) is measured. If the f/Vt ≤100 and SpO_2 ≥90%, patients pass the Zero CPAP Trial and proceed to an SBT. SBTs are conducted on T-piece or CPAP 0 cmH_20 and FiO_2 0.40 for 30-120 minutes.

Randomization Criteria

Patients who continue to meet the eligibility criteria, have passed the pressure support trial and have not met the Weaning Criteria or failed the Zero CPAP Trial or failed the SBT are eligible for randomization.

Adjusting PAV+

In the PAV+ strategy, the level of support (%Support or "gain") will be adjusted to target a respiratory muscle pressure (Pmus) within normal range, without evidence of respiratory distress. The Pmus target range is 5-10 cmH_2O, and the clinical targets are a RR <35 and Vte >5 mL/kg predicted body weight (PBW). At a minimum, patients will be assessed at least every 8 hours (or according to standard practice in each ICU if patient assessments are routinely performed more frequently than every 8 hours) to determine if adjustments need to be made to maintain Pmus within the target range.

Initial PAV+ gain will be set at 70% with PEEP at the setting being used prior to randomization. The inspiratory trigger will be set by the clinical team, and the expiratory trigger will be the default setting of 3L/min.

Details for adjusting settings for patients in the PAV+ strategy are summarized in the one-page ventilation algorithm for PAV+.

Adjusting PSV

In the PSV strategy, the level of pressure support will be adjusted according to routine clinical parameters, targeting a comfortable respiratory rate and tidal volume. At a minimum, patients will be assessed at least every 8 hours (or according to standard practice in each ICU if patient assessments are routinely performed more frequently than every 8 hours) to determine if adjustments need to be made

to maintain patient comfort and ensure adequate ventilation and oxygenation. The clinical targets are a RR 12-35 and Vte 5-10 mL/kg PBW.

The initial PSV setting will be between 10 and 20 cm H_2O or the level that the patient was on prior to randomization. The inspiratory trigger will be set by the clinical team, and the expiratory trigger will be the default setting of 25% of peak inspiratory flow.

Details for adjusting settings for patients in the PSV strategy are summarized in the one-page ventilation algorithm for PSV.

Ventilator adjustment common to both groups

We will aim to maintain pH in the range of 7.32-7.47. FiO_2 and PEEP will be titrated clinically to maintain a SpO_2 90-96%. Patients developing *Clinical Instability* or *Respiratory Distress* (see definitions below) that cannot be alleviated will be changed to A/C mode and reassessed within 24 hours for criteria to resume the assigned PAV+ or PSV strategy.

The definition of Respiratory Distress is the presence of at least 2 of the following:

1. SpO_2 <90%
2. Sustained (>5 min) respiratory rate >35 b/min
3. Heart rate >140 b/min or a sustained (>5 min) increase of 20% from baseline
4. Systolic blood pressure >180 or <80 mmHg and/or systolic BP changes >30% from baseline
5. Increased anxiety
6. Use of accessory muscles
7. Complaint of dyspnea
8. Diaphoresis

The definition of Clinical Instability is the presence of any 1 of the following:

1. Unstable hemodynamic status (SBP<80 mmHg) with or without vasoactive drug

2. Vasopressor requirements >0.5 ug/kg/min epinephrine/norepinephrine or equivalent

3. Active cardiac ischemia (dynamic ST changes on cardiac monitor or electrocardiogram)

4. Unstable arrhythmias (HR >140 or <50) with clinical signs of low cardiac output or SBP<80 mmHg

5. Uncontrolled hypertension (SBP>180 mmHg)

6. Abrupt decrease in the level of consciousness (RASS -4 or -5 or SAS 1 or 2)

7. Dangerous agitation (RASS +4 or +3 or SAS 7)

8. Metabolic (or mixed) acidosis with pH <7.32

9. Emergency situation that merits return to full ventilation (A/C) according to best clinical judgement

Weaning and Extubation

Weaning will be conducted in the same way in the two arms. The PSV level or the PAV+ %Support will not be used to assess readiness to wean. At a minimum, patients in both arms will be assessed at least once daily for Weaning Criteria (figure XX) and will undergo the weaning trials at least once daily if criteria at each stage of weaning are met. However, additional screening, additional pre-SBT readiness assessments, or additional SBTs may be performed at any time at the physician's discretion or as per institutional standard.

Patients who meet the Weaning Criteria will undergo an SBT readiness assessment by changing the ventilator to CPAP 0 and FiO_2 0.40 (Zero CPAP Trial). The f/Vt ratio will be assessed after 2 minutes. If the ratio is greater than 100, SpO_2 < 90% or there is clinical instability, the patient has failed this assessment and will be returned to the previous ventilator settings. If the patient passes, we will perform a spontaneous breathing test (SBT). The SBT will be performed on a t-piece, or tracheostomy

mask for patients with tracheostomy, with FiO_2 0.40. The SBT will be continued for 30 to 120 minutes. The SBT will be considered a failure if respiratory distress, clinical instability or increased somnolence (with elevated pCO_2 or pH < 7.32) occur. Patients who fail the SBT will be placed back on the previous ventilator settings. Patients who pass the SBT should be assessed for extubation based on the level of consciousness and strength of cough as judged by the clinical team. Patients who do not meet extubation criteria will be returned to ventilator settings according to their assigned algorithm. Justification must be provided in the case report form if patients meet extubation criteria, but extubation is deferred beyond 24 hours.

Sedation and other co-interventions

Guidelines for the administration of sedation and analgesia are provided, with a strong recommendation for using the lowest possible dose of sedating drugs in both arms (or none at all) as required to keep the patient calm and cooperative, avoiding over-sedation whenever possible. When sedation is necessary, we recommend assessing analgesia and intervening with appropriate pharmacological measures prior to administering sedatives. We will record the daily doses of sedatives, analgesics, and neuroleptic medications administered to the patient.

The assigned ventilation strategy may be interrupted for patient transfers away from the ICU. Non-invasive ventilation may be used post-extubation at the discretion of the treating clinician. Tracheostomies may be performed when clinically indicated.

Patients will continue on the assigned ventilation strategy until successful liberation from invasive MV, death, or 90 days post randomization, whichever comes first. All intubations and extubations (or for patients with tracheostomy, all disconnections lasting >48 hrs and reconnections following 48 hrs off

ventilator) will be tracked from date of hospital admission to day 90 or date of death. Date of the last follow-up will be 90 days post-randomization, when vital status will be recorded.

Trial Management

Study Oversight and Data Management

Lawson Health Research Institute (Lawson) in London, Ontario, Canada will oversee all contracts, trial insurance and financial disbursements.

The Applied Health Research Centre (AHRC) in Toronto, Ontario, Canada will serve as the Coordinating Centre (CC) for the study. Study site personnel will enter all data into electronic case report forms created in Medidata RAVE by the CC. The data is housed securely at St. Michael's Hospital in Toronto, Canada and appropriate security measures will be in place. Investigators will maintain and retain appropriate source and study documentation.

The Executive Committee (EC) will consist of the lead investigators (KJB, LB, KEAB, CM, JM, YS) with support from the study statisticians (KT, FZ, MLR) and the AHRC coordination centre. The executive committee will oversee all aspects of the study including implementation of all policies and the daily operations. The EC will meet weekly during the planning phase of the trial and bimonthly thereafter.

Study Monitoring

The coordinating centre will contact participating sites during the study to follow the recruitment and the return of study documents (e.g. copy of screening/enrollment log forms), and to address any issues or questions from the sites. The coordinating centre will also conduct site monitoring visits using a risk-based approach. A combination of centralized, on-line remote, and on-site monitoring activities will be used to ensure the quality of the data captured, the study operations, and the safety of patients.

Source data verification on critical data elements will be performed on a selection of the participants by comparing the data in the patient's files (source documents) with data in the CRF and will be conducted as per the Sponsor-approved Monitoring and Quality Plan. Electronic CRF will not constitute source documentation and data entered in the CRF must be traceable to an original source record (electronic or paper) either as part of the electronic database or in the patient's file. For selected patients, the presence of a signed written informed consent as well as compliance with inclusion and exclusion criteria will be checked.

Safety

Notwithstanding the safety measures provided within the ventilation algorithm, in the event of an emergency, the site PI and/or attending physician will ensure that best medical judgment is used to protect the life or well-being of the participant, which may include deviations from the protocol. The safety and welfare of participants in the study takes absolute precedence over the evaluation goals and objectives of the study.

The target population for this trial is critically ill patients with a high baseline risk of complications and death. We expect that some patients may die during the study. If the patient dies, the site investigator is required to complete a SAE assessment in their source documents. A death due to a decision to withdraw life support, or a decision to not re-intubate post extubation, or a change in the goals of care, does not constitute a serious adverse event and does not need to be reported as such. We are interested in reviewing Serious Adverse Events that may be related to the study interventions, rather than adverse events that occurred due to the underlying critical illness.

It is the responsibility of the site PI to ensure all SAE's are assessed to determine causality, that is, to determine the likelihood that the SAE was caused by a study intervention. The site PI will ask the

attending physician caring for the patient when the SAE occurred to assist with establishing or assessing causality. A SAE can be considered unrelated, unlikely related, possibly related, probably related, or definitely related to the study intervention, according to the definitions provided in the protocol. Cases where causality is “unknown”, or the site PI and attending physician have disagreement, or doubt, regarding whether an SAE has “unlikely” relation vs. “possible” relation to the study intervention, the site PI is mandated to contact one of the study co-PIs/sponsors to discuss and adjudicate the SAE.

An independent Data Safety Monitoring Board (DSMB) will review each Serious Adverse Event for which a possible, probable, or definite relationship to the study intervention exists. The DSMB will review safety reports biannually. The DSMB will have the ability to request additional safety analyses and make recommendations about the safe conduct of the trial. The DSMB Chair will review all serious adverse events classified as probably or definitely related to enrollment in the trial within 7 days and communicate directly with the co-principal investigators, who in turn will communicate this information to the Executive Committee.
