# Supplementary material for: Study protocol for a randomized controlled trial of Proportional Assist Ventilation for Minimizing the Duration of Mechanical Ventilation: the PROMIZING study
Source: Trials. 2023 Mar 27;24:232. doi: 10.1186/s13063-023-07163-w (PMC10041480; doi:10.1186/s13063-023-07163-w)

PSV VENTILATION STRATEGY

Assess at least once daily while on A/C for Criteria for Switching from A/C to PSV

INITIATION OF PSV

Ventilator Settings:
PS level: 10-20 cmH_2O or at previous level
Inspiratory trigger: per clinician
Expiratory trigger: 25% of peak flow$_i$
PEEP: at previous level
FiO_2:at lowest level required to keep SpO_2 90-96%

Alarm Settings:
Paw max: per clinician
RR max: 38 b/min
Vte max: 12 mL/kg of PBW
Vte min: 0 mL
VE max: 20 L/min
VE min: 5 L/min
Apnea setting: 20 s

Assess at least every 8 hours while on PSV to determine if adjustments are needed to maintain Target Range

**Repeat blood gas q 30 min as needed to confirm pH 7.32-7.47*

†Best clinical judgement should always prevail in setting of deterioration, with frequent reassessment until stable

ALGORITHM TO ADJUST PSV BASED ON CLINICAL PARAMETERS

No Respiratory Distress:
Patient appears comfortable
RR 12-35 and Vte 5-10 mL/kg PBW

→ **Target range achieved**.
No adjustments required unless one of the following occurs:

→ RR <12 or >35 or Vte <5 or >10 mL/kg PBW:
Obtain arterial blood gas.
No adjustments required unless:

→ Respiratory alkalemia
→ Treat any apparent cause of hyperventilation (eg. Pain, anxiety, metabolic acidosis)
→ Decrease PS in steps of 2-3 cmH_2O until Vte <8 mL/kg PBW and/or pH≤7.47

→ Respiratoryacidemia or **Respiratory Distress** (see definition)
→ Increase PS in steps of 2-3 cmH_2O until respiratory distress resolves and pH ≥7.32 (confirm with repeat blood gas*****)

Respiratory Distress with ≥2 signs:
See definition

→ Treat any apparent cause of respiratory distress (eg. secretions, bronchospasm, pulmonary edema)
→ Increase FiO_2 and/or PEEP as needed to keep SpO_2>90%
→ Increase PS in steps of 2-3 cmH_2O until respiratory distress resolves and pH ≥7.32 (confirm with repeat blood gas*****)
→ Switch to A/C mode if ≥ 1 of:
PS >20 cmH_2O
PS + PEEP > 30 cmH_2O
FiO_2>60% required for SpO_2 ≥90%
Clinical instability (see definition)**†**
Unable to trigger vent (NMB/sedation)
→ *Reassess within 24 hours for Criteria for Switching from A/C to PSV*

Assess at least once daily while on PSV for Criteria to Initiate Weaning; Attempt to wean FiO_2 to 0.40 and PEEP to ≤8 cmH_2O

ALGORITHM FOR WEANING

Criteria to Initiate Weaning:
SpO_2 ≥90%
FiO_2 ≤0.40
PEEP ≤8 cmH_2O
pH ≥ 7.32
vasopressors no higher than norepinephrine 0.1 ug/kg/min

→ Pre-SBT readiness assessment:
CPAP 0 cmH_2O and FiO_2 0.40 for 2 min;
assess f/Vt

→ f/Vt≤100 → SBT: t-piece, FiO_2 0.40 for 30 min → Pass SBT: assess for extubation / Fail SBT

→ f/Vt >100 → Return to PSV
Reassess within 24 hr

PAV+ VENTILATION STRATEGY

***Assess at least once daily** while on A/C for Criteria for Switching from A/C to PAV*

INITIATION OF PAV

Ventilator Settings:

IBW (Ideal Body Weight): Correct PBW (Predicted Body Weight

Tube Type: ET or Trach

PAV %support/gain:70%

Inspiratory trigger: per clinician

Expiratory trigger (Esens): 3 L/min

PEEP: at previous level

FiO_2: at lowest level required to keep SpO_2 90-96%

Alarm Settings:

Paw max: 40 cmH_2O

RR max: 38 b/min

Vte max: 12 mL/kg of PBW

Vte min: 0 mL

VE max: 20 L/min

VE min: 5 L/min

Apnea setting: 20 s

***Assess at least every 8 hours** while on PAV to determine if adjustments are needed to maintain Target Range*

**Repeat blood gas q 30 min as needed to confirm pH 7.32-7.47*

†Best clinical judgement should always prevail in setting of deterioration, with frequent reassessment until stable

ALGORITHM TO ADJUST PAV BASED ON PHYSIOLOGIC AND CLINICAL PARAMETERS

No Respiratory Distress: Calculate $P_{mus.Peak}$

- $P_{mus,Peak}$ <5 cmH_2O → Decrease gain in steps of 10% until $P_{mus,Peak}$ 5-10 cmH_2O
- $P_{mus,Peak}$ 5-10 cmH_2O → **Target range achieved:** No adjustments required unless:
  - RR <12 or >35 or Vte <5 or >10 mL/kg PBW: Obtain arterial blood gas. No adjustments required unless:
    - Respiratory alkalemia → Treat any apparent cause of hyperventilation (eg. Pain, anxiety, metabolic acidosis) → Decrease gain in steps of 10% until Vte <8 mL/kg PBW and/or pH≤7.47
    - Respiratory acidemia or **Respiratory Distress** (see definition) → Increase PEEP in steps of 2 cmH_2O (see right)
- $P_{mus,Peak}$ >10 cmH_2O → Increase the gain in steps of 10% until $P_{mus,Peak}$ 5-10 cmH_2O

Respiratory Distress with ≥2 signs: See definition

→ Assess PAV+ Resistance (R) and Compliance (C) measurements. Treat any apparent cause of increased R (eg. secretions, bronchospasm) or decreased C (eg. pulmonary edema)

→ **Increase PEEP in steps of 2 cmH_2O** until Compliance no longer increases

→ Increase FiO_2 as needed to keep SpO_2>90%

→ Increase PAV %support/gain in steps of 10% until respiratory distress resolves and pH ≥7.32 (confirm on blood gas*****)

→ Switch to A/C mode if ≥ 1 of:
PAV gain>85%
FiO_2>60% required for SpO_2 ≥90%
Clinical instability (see definition)**†**
Unable to trigger vent (NMB/sedation)

→ *Reassess within 24 hours for Criteria for Switching from A/C to PAV*

***Assess at least once daily** while on PAV for Criteria to Initiate Weaning; Attempt to wean FiO_2 to 0.40 and PEEP to ≤8 cmH_2O*

ALGORITHM FOR WEANING

Criteria to Initiate Weaning:
SpO_2 ≥90%
FiO_2 ≤0.40
PEEP ≤8 cmH_2O
pH ≥ 7.32
vasopressors no higher than norepinephrine 0.1 ug/kg/min

→ Pre-SBT readiness assessment: CPAP 0 cmH_2O and FiO_2 0.40 for 2 min; assess f/Vt

- f/Vt≤100 → SBT: t-piece, FiO_2 0.40 for 30 min → Pass SBT: assess for extubation / Fail SBT
- f/Vt >100 → Return to PAV *Reassess within 24 hr*

Weaning /SBT Methods for PROMIZING

Pre-SBT readiness assessment (CPAP Trial)

1. With patient's ETT connected to the ventilator circuit, change the ventilator mode to CPAP at 0 cmH_2O and FiO_2 0.40 (**on PB 840 ventilator, choose MODE: SPONT and then choose Spontaneous Type NONE, then set PEEP to 0 and FiO_2 to 40%)**
2. Monitor patient for 2 min
3. On the ventilator, assess the f/Vt ratio after 1-2 minutes at 0 cmH_2O CPAP:
4. **FAIL**: If the f/Vt >100, or SpO2<90% or Clinical Instability, patient has failed the CPAP trial. Resume ventilation on settings used immediately prior to the CPAP test.
5. **PASS**: If the f/Vt is ≤100, and SpO_2 ≥90%, proceed to SBT

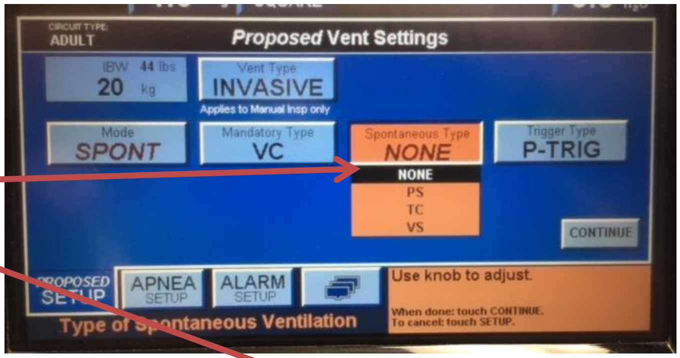

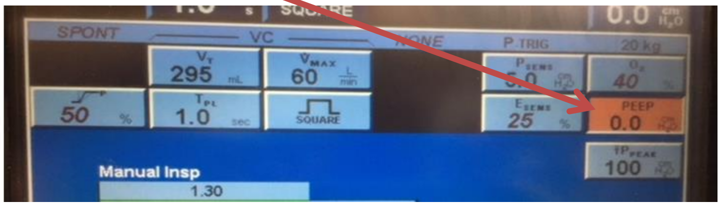

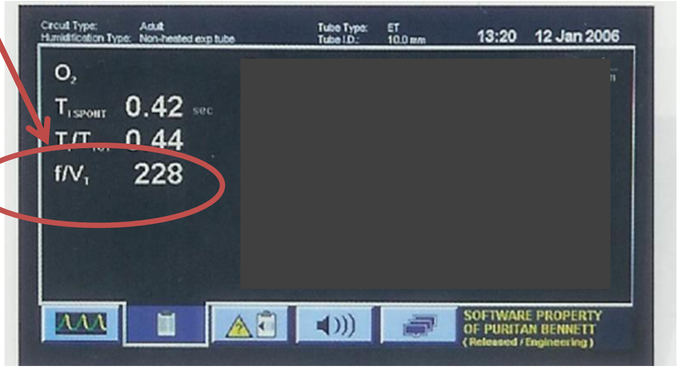

SBT

1. Disconnect endotracheal tube from ventilator circuit and place patients on T-piece* with FiO_2 0.40. (For patients with tracheostomy, place patients on tracheostomy mask trial with FiO_2 0.40)
2. Monitor patient for 30-120 minutes:
3. **FAIL/ SBT Termination**: Terminate the SBT and return patient to ventilator settings according to assigned algorithm if at any time one of the following occur:
    a. Respiratory Distress
    b. Clinical Instability
    c. Increased somnolence with elevated pCO_2 and/or pH<7.32
    d. Desaturation with SpO2 <90% on FiO2 0.40
4. **PASS:** Patients will be considered to have passed the SBT if, after a minimum of 30 minutes and maximum of 120 minutes, none of the failure/SBT termination criteria have occurred.
5. **Extubation/Disconnection:** Proceed to evaluate patients with endotracheal tube for extubation criteria. Patients with tracheostomy who pass their initial SBT may remain off ventilator until they develop failure criteria. Tracheostomized patients who fail their initial SBT within 120 minutes may undergo gradual increments in tracheostomy mask trials according to local practice.

**for the purposes of this study, the Sponsor considers SBT performed with patients connected to ventilator on flow-by with CPAP of 0 cmH_2O and FiO_2 0.40 to be equivalent to SBT performed on T-piece with FiO_2 0.40.*

T-Piece

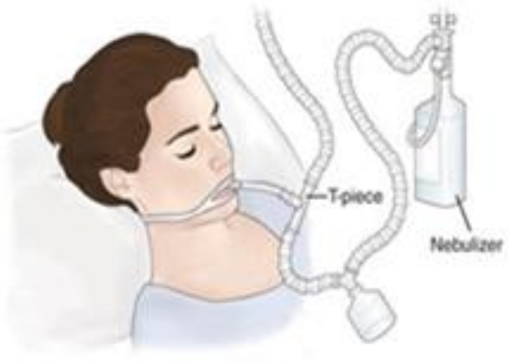

Tracheostomy Mask Trial

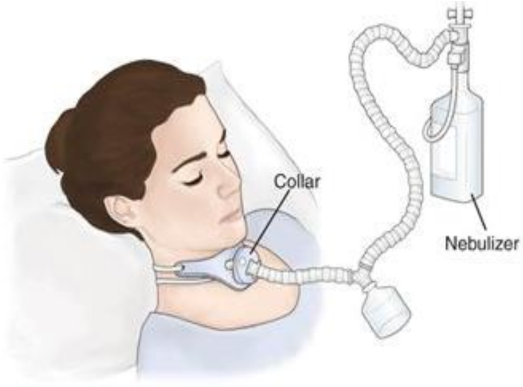

Supplement: Supplementary file 2 — Additional file 2. PAV_PSV ventilation algorithms. [file 13063_2023_7163_MOESM2_ESM.pdf]
