# Supplementary material for: Study protocol for a randomized controlled trial of Proportional Assist Ventilation for Minimizing the Duration of Mechanical Ventilation: the PROMIZING study
Source: Trials. 2023 Mar 27;24:232. doi: 10.1186/s13063-023-07163-w (PMC10041480; doi:10.1186/s13063-023-07163-w)
Supplement: Supplementary file 3 — Additional file 3. Rationale for Changes to Protocol. [file 13063_2023_7163_MOESM3_ESM.pdf]

Rationale for Changes to Protocol and Primary Outcome

Brief Summary of Changes:

1. We have revised the primary outcome to be time from randomization to successful extubation and assigned ventilator-free days at day 14, 21 and 28 post-randomization as a secondary outcome.

2. Based on this new outcome, we have increased the minimum planned sample size from 512 to 558.

3. We have updated the funding information to reflect a new grant and extended the planned study period accordingly.

4. We have modified subgroups for a priori analyses.

5. We have added and modified some study inclusion/exclusion criteria.

6. We have revised the statistical analysis plan based on the new primary outcome.

7. Various formatting changes for clarity.

Rationale for Changes to the Protocol:

1. We have revised the primary outcome to be time from randomization to successful extubation, which was previously a secondary outcome, and assigned all ventilator-free days calculations (day 14, 21 and 28 post-randomization) as secondary outcomes (VFDs was previously the primary outcome):

We have determined from preliminary analysis of aggregate, blinded data (planned a priori to re-estimate sample size after first 120 patients randomized) and discussion of statistical considerations that ventilator-free days is not the most appropriate primary outcome for this study. There are **four main reasons** for this decision. First, VFDs at day 21 was not capturing the outcome of a substantial proportion of patients still ventilated at day 21 after enrollment in the study. **This was not expected when the study was planned**, so we had to change this outcome. In fact, no data on time to extubation starting at the time of first attempt to separation was available at the time this study was designed. The WIND Study, an epidemiological study on weaning outcome, was not published until 2017, and provided new data previously not known, showing that patients who fall into weaning groups 2 and 3 have high incidence of patients not weaned from MV or having died within 28 days of the first separation attempt (Figure A1). Second the distribution of the VFD data has two peaks since there are a **large number of patients who have zero VFDs** (Figure A1). It is therefore unclear how the new treatment can have a meaningful impact on this distribution. Third, VFDs is difficult to understand. For stakeholders this means it is difficult to apply the data and understand the implications. For families and patients this means it can cause confusion. Last, VFDs is not a patient-centred value. A value of zero might mean the

patient was ventilated during the entire study period, then was successfully extubated and discharged; but it might also mean the patient died within the study period. As indicated by a patient advisor to our study, these two outcomes are certainly not of equal value to the patient and should be distinctly identified in the data. **This issue has been raised repeatedly in the medical literature since the start of our study**.

We propose to switch the primary outcome measure to be “time to successful liberation”, which was previously a secondary outcome measure. This value is easier to understand for patients, families and other stakeholders and will capture all patients. This patient-centred value makes it completely clear how long a patient was ventilated for. A single day of difference in this value is important to patients.

We will continue to record VFDs at 14, 21- and 28-days post-randomization as a secondary outcome measure. The remaining secondary outcome measures are unchanged from the previous version of the protocol.

A1.a

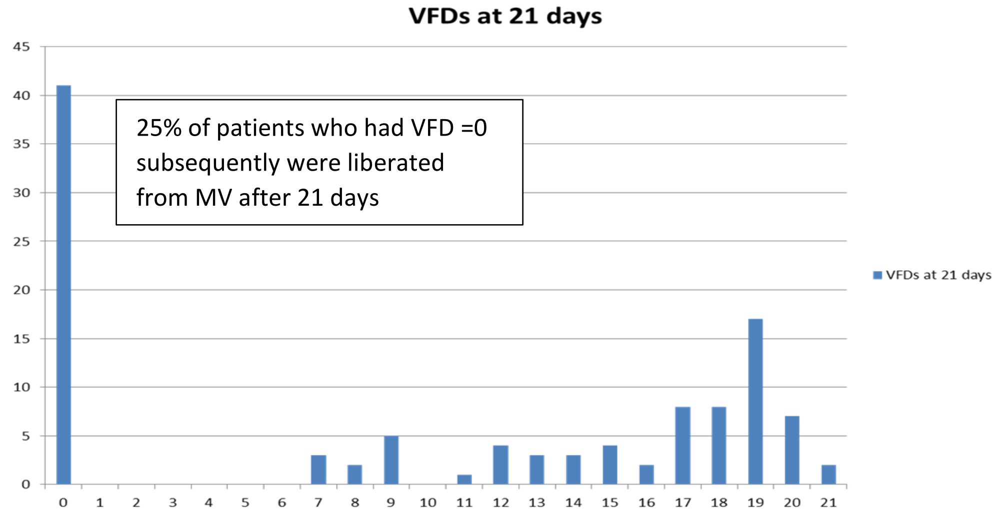

A1.b

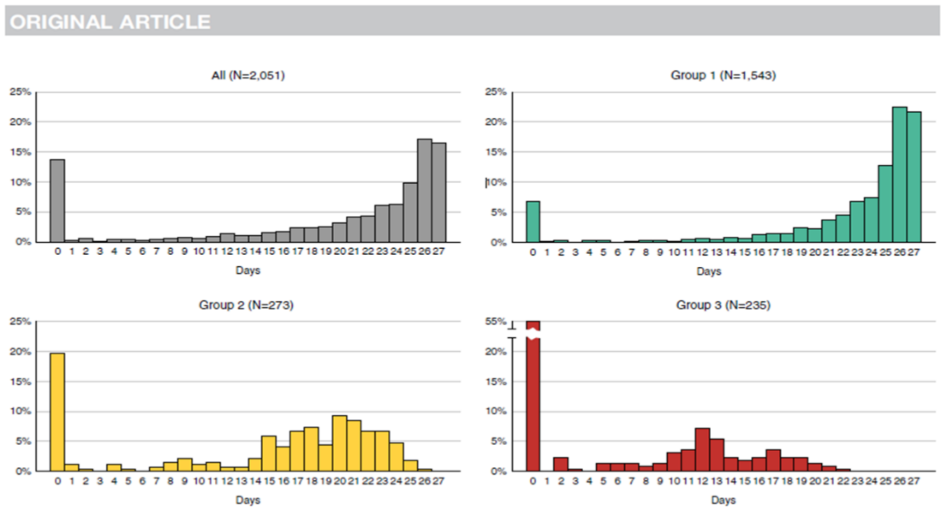

Figure 3. Distribution of ventilator-free days at Day 28 according to weaning group. This figure shows for each group and for the whole population the proportion of patients achieving each number of ventilator-free days from 27 to 0. Ventilator-free days are defined by 28 minus the total number of days with invasive mechanical ventilation. Nonsurvivors were considered as having 0 ventilator-free days.

Figure A1: PROMIZING VFD distribution from aggregate, blinded data (a) is similar to groups 2 and 3 of the WIND study (b) (AJRCCM 2017)[i], **unknown at the time of designing the study** (no data starting at the time of first attempt to separation was available at the time this study was designed).

2. We have increased the minimum planned sample size from 512 to 558.

As planned a priori, we used blinded, aggregate data from the first group of patients to re-estimate our sample size. Based on this analysis, detailed in the revised protocol section 12.1, we determined we will require a minimum sample size of 558 patients (279 per group). We also secured additional funding from a 2018 Fall CIHR grant, allowing us to extend the duration of the study and thus making the increased sample size feasible.

A table of sample sizes for time from randomization to first successful extubation/liberation is shown below. These calculations incorporate death by treating death as censoring. The current hazard rate for death is used in this calculation. The final column is obtained by dividing the "Total N" column by 0.95 to account for the 5% dropout. Assuming this increase is sufficient to observe the required number of events in the presence of the drop-out, it should be a reasonable target. All calculations are using 80% power and 2-sided Type I error of 5%. Exponential survival is assumed for the purposes of hazard calculations. The expected median time to extubation in PAV is determined from the PSV value by converting to the hazard scale, multiplying by the hazard ratio and converting back to time.

Median Time (PSV)	Median Time (PAV)	Difference	Hazard Ratio	Required Events	Total N	Total N (5% lost)
7.40	6.17	1.23	1.20	944	1095	1153
7.40	5.92	1.48	1.25	630	729	768
7.40	5.69	1.71	1.30	456	526	554
7.50	6.25	1.25	1.20	944	1097	1155
7.50	6.00	1.50	1.25	630	731	770
7.50	5.77	1.73	1.30	456	527	555
7.55	6.29	1.26	1.20	944	1098	1156
7.55	6.04	1.51	1.25	630	731	770
7.55	5.81	1.74	1.30	456	528	556
7.60	6.33	1.27	1.20	944	1099	1157
7.60	6.08	1.52	1.25	630	732	771
7.60	5.85	1.75	1.30	456	528	556
7.70	6.42	1.28	1.20	944	1101	1159
7.70	6.16	1.54	1.25	630	733	772
**7.70**	**5.92**	**1.78**	**1.30**	**456**	**529**	**557**

As planned a priori, we used aggregate, blinded data from the first 120 patients to re-estimate our sample size. Using a time to event analysis, median time to successful liberation in the entire cohort was 6.8 days. The minimum clinically important difference in time to successful liberation is deemed to be 1.0 day. Using a hazard ratio of 1.30, to demonstrate a reduction in the median duration of ventilation by 1.78 days (assuming 7.70 days versus 5.92), alpha of 0.05 (two-sided) and a power of 80%, requires 529 patients. Anticipating a maximum loss to follow-up (e.g. consent withdrawn) rate of 5%, 558 patients (279 per group) should be randomized in the study. Using a hazard ratio of 1.25, to demonstrate a reduction in the median duration of ventilation by 1.51 days would require770 patients (385 per group) to be randomized in the study. We anticipate being able to enroll a minimum of 558 patients within the planned enrolment period. If enrolment exceeds expectations, we will be powered to show a smaller difference between the 2 groups, which will still be clinically important. The enrolment period will continue until we have complete data on randomized participants and have attained the minimum number of required events in our study cohort.

3. We have updated the funding information to reflect a new grant and extended the planned study period accordingly.

We propose extending the anticipated study end date to accommodate recruitment of the increased sample size.

4. We have modified subgroups for analyses.

We have added "failed CPAP" and "failed weaning criteria" to the "failed SBT" subgroup. By comparing each of these criteria against time to successful extubation, we can better understand how each these values may allow prediction of how close a patient is to successful extubation.

5. We have added and modified some of study inclusion/exclusion criteria.

A11: We added additional screening exclusion criteria, summarized as item A11. We aim to exclude patients from this study who are unlikely to be liberated from the ventilator due to their underlying cardio-respiratory disease and are awaiting lung or heart transplant or other surgery. In this study, we are specifically interested in patients who are likely to be extubated. We had not initially identified this specific group as one which would be unlikely to be extubated.

B11: We relocated this enrollment deferral criteria in the protocol to make the flow more logical.

B12: We added additional enrollment deferral criteria, summarized as item B12. We have indicated that patients on ECMO should be deferred from enrolling in this study. We propose deferring enrollment of ECMO patients because it is possible for a patient to be extubated while still on ECMO; this would present a confounding variable in the data, since they are extubated but still on life support. Accordingly, we have now specified to wait until patients are no longer on ECMO before considering enrollment in the study.

C9: This was previously listed under pressure support criteria. We decided it was more efficient to apply this criterion to defer enrollment, rather than apply it after enrollment.

C11: This criterion has been removed. This criterion was redundant, as criteria B1 already states the patient must have the potential to trigger their own breaths. Furthermore, the patient's ability to trigger breaths will be assessed during the SBT, so this additional step is not necessary.

B9: We have now re-iterated point B9 on page 21 of the protocol. This is a secondary check, as it is possible for a patient to be enrolled and not randomized for a period of time. Thus, it would be possible that during this lag a patient may meet this exclusion criteria, at which point they should not be randomized to a treatment arm.

6. We have revised the statistical analysis plan.

We have modified our primary outcome measure and conducted analysis, as planned a priori, on the first group of patients. Thus, we have modified our statistical analysis plan to: 1) account for the change in primary outcome measure to be time to liberation and 2) describe the statistical analysis which we determined was optimal based on our preliminary analysis.

---

[i] Béduneau G, Pham T, Schortgen F, Piquilloud L, Zogheib E, Jonas M, Grelon F, Runge I, Nicolas Terzi, Grangé S, Barberet G, Guitard PG, Frat JP, Constan A, Chretien JM, Mancebo J, Mercat A, Richard JM, Brochard L; WIND (Weaning according to a New Definition) Study Group and the REVA (Réseau Européen de Recherche en Ventilation Artificielle) Network ‡. Epidemiology of Weaning Outcome according to a New Definition. The WIND Study. Am J Respir Crit Care Med. 2017 Mar 15;195(6):772-783. doi: 10.1164/rccm.201602-0320OC. PMID: 27626706.
